# Tannic acid supplementation in the diet of Holstein bulls: Impacts on production performance, physiological and immunological characteristics, and ruminal microbiota

**DOI:** 10.3389/fnut.2022.1066074

**Published:** 2022-11-16

**Authors:** Zuo Wang, Yuan Zhao, Xinyi Lan, Jianhua He, Fachun Wan, Weijun Shen, Shaoxun Tang, Chuanshe Zhou, Zhiliang Tan, Yanming Yang

**Affiliations:** ^1^College of Animal Science and Technology, Hunan Agricultural University, Changsha, Hunan, China; ^2^CAS Key Laboratory of Agro-Ecological Processes in Subtropical Region, National Engineering Laboratory for Pollution Control and Waste Utilization in Livestock and Poultry Production, Hunan Provincial Key Laboratory of Animal Nutrition and Physiology and Metabolism, Institute of Subtropical Agriculture, Chinese Academy of Sciences, Changsha, Hunan, China; ^3^Jiurui Biology and Chemistry Co., Ltd., Zhangjiajie, Hunan, China

**Keywords:** tannic acid, Holstein bulls, production performance, ruminal microbiota, inflammatory response

## Abstract

This study was conducted to evaluate the influences of supplementing tannic acid (TA) at different doses on the production performance, physiological and immunological characteristics, and rumen bacterial microbiome of cattle. Forty-eight Holstein bulls were randomly allocated to four dietary treatments: the control (CON, basal diet), the low-dose TA treatment [TAL, 0.3% dry matter (DM)], the mid-dose TA treatment (TAM, 0.9% DM), and the high-dose TA treatment (TAH, 2.7% DM). This trial consisted of 7 days for adaptation and 90 days for data and sample collection, and samples of blood and rumen fluid were collected on 37, 67, and 97 d, respectively. The average daily gain was unaffected (*P* > 0.05), whilst the ruminal NH_3_-N was significantly decreased (*P* < 0.01) by TA supplementation. The 0.3% TA addition lowered (*P* < 0.05) the levels of ruminal isobutyrate, valerate, and tumor necrosis factor alpha (TNF-α), and tended to (*P* < 0.1) increase the gain to feed ratio. The digestibility of DM, organic matter (OM), and crude protein, and percentages of butyrate, isobutyrate, and valerate were lower (*P* < 0.05), while the acetate proportion and acetate to propionate ratio in both TAM and TAH were higher (*P* < 0.05) than the CON. Besides, the 0.9% TA inclusion lessened (*P* < 0.05) the concentrations of glucagon and TNF-α, but enhanced (*P* < 0.05) the interferon gamma (IFN-γ) level and Simpson index of ruminal bacteria. The 2.7% TA supplementation reduced (*P* < 0.05) the intake of DM and OM, and levels of malondialdehyde and thyroxine, while elevated (*P* < 0.05) the Shannon index of the rumen bacterial populations. Moreover, the relative abundances of the phyla *Fibrobacteres* and *Lentisphaerae*, the genera *Fibrobacter* and *Bradyrhizobium*, and the species *Bradyrhizobium* sp., *Lachnospiraceae* bacterium RM29, and *Lachnospiraceae* bacterium CG57 were highly significantly (*q* < 0.01) or significantly (*q* < 0.05) raised by adding 2.7% TA. Results suggested that the TA addition at 0.3% is more suitable for the cattle, based on the general comparison on the impacts of supplementing TA at different doses on all the measured parameters.

## Introduction

Tannins are a class of natural polyphenolic compounds in plants with a strong capacity to form complexes with protein and carbohydrate fractions through the hydrogen bonds (1, 2). It has been revealed in previous studies that the addition of tannins can enhance the nitrogen utilization and decrease methane emission of ruminants (1, 3–6). By forming the tannin-protein complexes at the normal ruminal pH, tannins could protect the feed protein from the microbial decomposition and thus raise the amount of rumen undegradable protein (RUP) (2). Furthermore, it has been shown that the suppressing influences of tannins on methane production can be attributed to the declines in the abundances of ruminal methanogens and protozoa, as well as the decrease of microbial fiber degradation (1, 3, 5).

According to the structure and reactivity, tannins can be generally classified into condensed tannins (CTs) and hydrolysable tannins (HTs) (7). The bioactive effect of tannins on the metabolism of ruminants is dependent on their source, type, dosage, the basal diet, and the animal (3, 8–10). Since the synthesis of ruminal microbial protein could also be reduced, the rise in RUP might not certainly increase the net flow of the metabolizable protein (MP) into the small intestine by supplementing tannins (11). It was reported that the addition of CT from quebracho extract [0.4% of dry matter (DM)] improved the flux of RUP and MP, and the utilization of feed crude protein in beef steers fed a high-concentrate (87% of DM) ration with soybean meal as the true protein source (12). In contrast, Henke et al. (13) found that the apparent digestibility of dietary crude protein of dairy cows was lowered by the inclusion of CT from quebracho at 15 and 30 g/kg DM in a diet containing 34% concentrate.

In comparison to the CTs with the molecular weight of 1,900–28,000 Da, HTs are relatively smaller (500–3,000 Da). Besides, the affinity for protein of HTs is lower than that of the CTs, which makes the HTs more easily absorbed by the digestive tract and hence increases the probable toxicity to ruminants (14). However, Liu et al. (4) reported that the dietary supplementation of chestnut-originated HT at 15 and 30 g/kg DM caused no toxicity to the sheep. The production performance of beef cattle fed the high-forage ration supplemented with either 0.25% or 1.5% chestnut HT was unaffected compared with the cattle fed the control diet (14). Tannic acid (TA) is a typical HT comprised of 8–10 molecules of gallic acid per molecule of glucose, and has a simpler structure than the CTs (2). Yang et al. (5) observed that the dietary inclusion of TA at 6.5, 13.0, or 26.0 g/kg DM reduced the methane yield (L/kg DM intake) of cannulated adult bulls, and also decreased the digestibility of DM, organic matter, and crude protein to different extents. Adding TA at 16.9 g/kg DM decreased the urea excretion, as well as the N_2_O-N emission from the urine of steers (15). The antioxidant activity of TA has been validated through both chemical and cellular antioxidant assays (16). A dose-dependent rehabilitation of the superoxide dismutase (SOD) activity by TA in thioacetamide-treated rats was noticed previously (17). Ugur Calis et al. (18) also reported that the pretreatment of TA could reduce the oxidative stress of rats treated with monosodium glutamate by decreasing the malondialdehyde (MDA) level in the brain homogenate and enhancing the SOD activity in blood hemolysates. Thus, it could be inferred that TA might serve as an antioxidant additive for ruminants.

Most investigations on the effects of applying TA in the diet for ruminants has been basically focused on the methane emission and the nitrogen utilization (5, 15). Influences of TA inclusion at different doses on the physiological status including the oxidative stress and inflammatory response, as well as the detailed alterations of ruminal microflora in cattle remain somewhat uncertain. The ruminal microbiota is associated with the productivity and health of the host (19) and effects of tannins on rumen microorganisms have been reported in a few previous studies (20, 21). Therefore, it is of significance to gain deep insights into the impacts of supplementing TA at different levels on the ruminal microbiota of cattle, through the application of third-generation sequencing that targets the full-length 16S rRNA gene and provides higher accuracy and resolution of microbial populations compared to the precedent partial 16S rRNA gene sequencing (19, 22).

Therefore, we hypothesized that TA might exert more impacts on ruminants than what had been reported in previous studies. In the present study, we aimed to comprehensively investigate the influences of dietary TA addition at different doses on the production performance, physiological, oxidative and immunological parameters, and ruminal microflora of Holstein bulls, so as to broaden understandings on the effects of TA application in the ruminants industry.

## Materials and methods

### Animals, diets, and management

All procedures involving animals in this study were approved by the Animal Care Committee (approval number: 20200805), College of Animal Science and Technology, Hunan Agricultural University, Changsha, China. This experiment was conducted in a completely randomized design. Forty-eight fattening Chinese Holstein male cattle (initial mean ± SE; 588 ± 54.6 kg of body weight, and 15 ± 1 months of age) were randomly assigned to each of the four dietary treatments: the control group (the basal TMR ration, CON), the low-dose TA treatment (the basal ration supplemented with TA at 0.3% DM, TAL), the mid-dose TA treatment (the basal ration supplemented with TA at 0.9% DM, TAM), and the high-dose TA treatment (the basal ration supplemented with TA at 2.7% DM, TAH). The ingredients and nutritional compositions of the basal TMR diet are displayed in Table 1. The TA (purity 97%) was a commercial product extracted from the *Rhus chinensis* Mill, i.e., Chinese sumac (Jiurui Biology and Chemistry Co., Ltd., Zhangjiajie, China). Cattle in each treatment group were randomly allocated into 3 pens, with 4 cattle housed in each pen. All the cattle were fed *ad libitum* twice per day at 06:00 h and 16:00 h with free access to fresh water. The whole period of this experiment was 97 days, comprising 7 days of adaptation and 90 days of data and sample collection. The total feed intake of the 4 bulls in each pen was recorded daily.

**TABLE 1:** Components and nutritional composition of the basal TMR ration.

Ingredients^a^, g/kg DM		Nutritional composition^b^, g/kg DM	
Wheat bran	269	NE^m^, Mcal/kg DM	1.4
Rice straw	229	NE^g^, Mcal/kg DM	0.8
Maize silage	60	OM	923.5
Peanut hull	100	CP	141.3
Soybean meal	148	NDF	422.6
Corn meal	100	ADF	268.1
DDGS	20	EE	52.3
Tofu pulp	20	Ca	7.6
Premix	25	P	4.3
CaHCO_3_	13		
NaHCO_3_	10		
NaCl	6		

^a^DM, dry matter; DDGS, distillers’ dried grains with soluble; Every 1 kg of premix contained 400 mg of Zn, 100 mg of Cu, 200 mg of Fe, 3,600 mg of Mg, 350 mg of Mu, 96 mg of Cr, 4.0 mg of Co, 50 mg of Se, 500 mg of Lysine, 500 mg of Methionine, 2500000IU of vitamin A, 100000IU of vitamin D3, and 4000IU of vitamin E.

^*b*^NEm, net energy for maintenance, calculated according to the China Feeding Standard of Beef Cattle (NY/T815-2004).

NEg, net energy for gain, calculated according to the China Feeding Standard of Beef Cattle (NY/T815-2004). OM, organic matter; CP, crude protein; NDF, neutral detergent fiber; ADF, acid detergent fiber; EE, ether extract.

### Sample collection

The TMR feed and the leftover of each pen were sampled every 5 days, while the feces samples from each pen were collected twice daily from the 91 to 97 d, by using previously described methods (23). For the sampling of rumen fluid and blood, two cattle were firstly randomly selected from each pen. Afterward, the samples of rumen fluid and blood were thence taken from those chosen cattle on 37, 67, and 97 d, respectively. The rumen fluid from the central rumen was collected 4 h after morning feeding through the oral cavity using an oral stomach tube as reported by Shen et al. (24). Briefly, after discarding the initial 150 mL of rumen liquid, the subsequent 150 mL was obtained and then filtered through 4 layers of gauze under a continuous CO_2_ stream. The blood samples were firstly taken using vacuum tubes through the coccygeal vein 4 h after morning feeding, subsequently placed at room temperature for 30 min, and finally centrifuged at 1,500 × g for 10 min at 4°C to obtain the serum samples. As for the plasma samples, blood was collected using a 10 mL heparinized tube, followed by the centrifugation at 3,500 × g for 15 min at 4°C. All the samples were instantly frozen in liquid nitrogen and stored at −80°C until further analysis.

### Chemical and biochemical analysis

By referring to the protocols of AOAC (25), the dry matter (DM; method 930.15), ash (method 942.05), crude protein (CP; method 2001.11), ether extract (EE, method 920.39), neutral detergent fiber (NDF; method 2002.04), and acid detergent fiber (ADF; method 973.18) in the feed and feces were analyzed. The contents of calcium (Ca) and phosphorus (P) in the basal ration were assessed as described previously (23, 26). The measurement for pH and the concentrations of ammonia nitrogen (NH_3_-N) and volatile fatty acid (VFA) of the rumen fluid was performed using methods depicted in precedent studies (19, 27). The acid-insoluble ash was used as the internal marker to estimate the apparent digestibility of nutrients (28).

The amount of lipopolysaccharide (LPS) endotoxin in the rumen fluid and plasma, and the levels of the inflammatory mediators in the serum were evaluated as previously described (26). As to the assessments of biochemical blood parameters, a Roche Cobas automatic biochemistry analyzer (c311, Roche Ltd., Basel, Switzerland) and corresponding specific kits (Roche Ltd., Basel, Switzerland) were used, according to the manufacturer’s instructions and prior report (29).

### Deoxyribonucleic acid isolation, polymerase chain reaction amplification, and full-length 16S rRNA gene sequencing

The extraction of total genomic deoxyribonucleic acid (DNA) from the rumen liquid samples was performed using a phenol-free bead-beating method as previously described (30). The quantity and purity of isolated DNA were measured on a ND-1000 spectrophotometer (NanoDrop Technologies Inc., Wilmington, USA). Afterward, the full-length bacterial 16S rRNA genes were amplified based on the genomic DNA isolated from rumen fluid with universal primers 27F (5′- AGRGTTTGATYNTGGCTCAG-3′) and 1492R (5′- TASGGHTACCTTGTTASGACTT-3′) with barcode, as depicted in precedent studies (19, 31). All polymerase chain reaction (PCR) reactions were carried out with the TransStart FastPfu DNA Polymerase (TransGen Biotech, Beijing, China), in accordance with the previously reported procedures (19). All the DNA samples were amplified in duplicates, with three wells serving as the negative control in each run. After the duplicate PCR products were mixed, the correct sizes of PCR products and the absence of signal from negative controls were subsequently examined through the agarose gel electrophoresis. The 16S rRNA gene amplicons with barcode were pooled in equidensity ratios, followed by the purification with QIAquick Gel Extraction Kit (QIAGEN, Germantown, USA). The amplicon sequencing library was constructed using the SMRTbellTM Template Prep Kit (Pacific Biosciences, Menlo Park, USA) following the manufacturer’s protocol, and the quality of the sequencing library was subsequently assessed on the Qubit 2.0 Fluorometer (Thermo Fisher Scientific, Waltham, USA). The sequencing of the qualified library was conducted on the PacBio Sequel II platform (Pacific Biosciences, Menlo Park, USA) and single-end reads were generated.

### Bioinformatic analysis

The bioinformatics analysis in the current trail was conducted with the aid of the NovoMagic Cloud (Novogene Co., Ltd., Beijing, China). Firstly, the circular consensus sequencing (CCS) reads recognition, CCS reads quality filtering, and chimera sequence removal were conducted using protocols in our prior reports (19, 32). Afterward, the processes including the operational taxonomic unit (OTU) clustering, OTU taxonomy assignment, and OTU abundance normalization were successively performed as described precedently (19, 32–34). The QIIME (V1.9.1) and R software (V3.1) were used to fulfill the analysis of the Alpha diversity and Beta diversity, and the function prediction of Tax4Fun was conducted by using methods in prior reports (19, 32–35). All the raw sequences in this trial were deposited to the sequence read archive (SRA) of the NCBI database under the accession number PRJNA770103.

### Statistical analysis

The PROC MIXED procedure of SAS (V9.4, SAS Institute Inc., Cary, USA) was used to evaluate the effects of supplementing TA at different doses on nutrient intake, production performance, nutrient digestibility, rumen fermentation parameters, and physiological and immunological indexes. For the statistical analysis of nutrient intake, performance, and nutrient digestibility, the statistical model included dose, sampling date, and their interaction as the fixed effects, with sampling date as the repeated measurement and individual pen as the experimental unit. As to the statistical analysis of rumen fermentation profiles, blood physiological and immunological indexes, and the Alpha diversity indices, the statistical model included dose, sampling date, and their interaction as the fixed effects, with sampling date as the repeated measurement and individual animal as the experimental unit. Linear and quadratic effects of dose were analyzed using orthogonal polynomial contrasts. Least squares means are reported throughout the text. Statistical difference was, respectively, declared as significant or highly significant at *P* < 0.05 or *P* < 0.01, while trend was discussed at 0.05 < *P* ≤ 0.10. The Metastats analysis was employed in the multiple comparison of bacterial relative abundances across treatments through the permutation test, and the *q*-values [i.e., the false discovery rate (FDR) adjusted *P*-values] were adopted (36). The significant difference or highly significant difference were thence considered at *q* < 0.05 or *q* < 0.01, respectively.

## Results

### Nutrient intake, performance, and apparent nutrient digestibility

Supplementing TA linearly (*P* < 0.01) reduced the DM and OM intake, and the DM and OM intake of the cattle in the TAH group was both significantly lower (*P* < 0.05) than that of the bulls in the CON group (Table 2). The average daily gain (ADG) tended to (*P* < 0.1) decline as the TA dose rose. The addition of TA at 0.3% DM tended to (*P* < 0.1) raise the ratio of gain to feed of the cattle, when compared to the CON. The apparent digestibility of DM (quadratic, *P* < 0.01), OM (linear, *P* < 0.01), and CP (linear, *P* < 0.01) of the cattle decreased in response to the supplementation of TA.

**TABLE 2:** Effects of tannic acid at different supplemental levels on nutrient intake, performance, and apparent nutrient digestibility of Holstein bulls.

Items^1^	Treatments^2^				SEM^3^	*P*-value^4^		
	CON	TAL	TAM	TAH		Dose	L	Q
**Nutrient intake, kg/d**								
DM	13.6^a^	13.0^ab^	12.7^ab^	12.1^b^	0.28	0.038	0.009	0.266
OM	12.8^a^	12.1^ab^	12.0^ab^	11.4^b^	0.26	0.029	0.008	0.242
CP	1.77	1.77	1.74	1.65	0.052	0.392	0.105	0.858
EE	0.47	0.49	0.53	0.49	0.025	0.431	0.815	0.125
NDF	6.22	6.17	6.21	5.65	0.160	0.099	0.023	0.415
ADF	4.08	4.07	4.14	3.72	0.124	0.143	0.043	0.292
**Performance**								
ADG, kg/d	1.09	1.28	1.09	0.96	0.106	0.113	0.067	0.696
G:F	0.08	0.10	0.09	0.08	0.007	0.079	0.176	0.381
**Apparent nutrient digestibility, %**								
DM	74.5^a^	73.1^a^	69.3^b^	67.6^b^	0.63	0.001	<0.0001	0.006
OM	69.7^a^	69.2^a^	65.1^b^	63.3^b^	1.00	0.004	0.001	0.089
CP	66.8^a^	65.5^a^	60.7^b^	57.9^b^	1.09	0.001	0.001	0.054
EE	64.3	64.2	63.5	58.2	1.92	0.147	0.031	0.636
NDF	70.3	69.8	67.8	67.8	0.90	0.168	0.077	0.176
ADF	61.1	61.9	60.6	57.8	1.43	0.264	0.068	0.739

^1^DM, dry matter; OM, organic matter; CP, crude protein; EE, ether extract; NDF, neutral detergent fiber; ADF, acid detergent fiber; ADG, average daily gain; G:F, the ratio of gain to feed.

^2^CON, control group; TAL, low-dose tannic acid treatment, 0.3% DM; TAM, mid-dose tannic acid treatment, 0.9% DM; TAH, high-dose tannic acid treatment, 2.7% DM.

^3^SEM, standard error of means for treatments.

^4^L, linear effect of the tannic acid dose; Q, quadratic effect of the tannic acid dose.

^a,b^Means within a row for treatments (*P* < 0.05).

### Rumen fermentation characteristics

The increasing proportion of TA decreased the densities of NH_3_-N (linear, *P* < 0.01), total volatile fatty acid (TVFA) (quadratic, *P* < 0.01), butyrate (linear, *P* < 0.05), isobutyrate (linear, *P* < 0.01), and valerate (linear, *P* < 0.01), whilst elevated the acetate portion (quadratic, *P* < 0.05) and the acetate to propionate ratio (quadratic, *P* < 0.01) to different extents (Table 3).

**TABLE 3:** Effects of tannic acid at different supplemental levels on rumen fermentation characteristics of Holstein bulls.

Items^1^	Treatments^2^				SEM^3^	*P*-value^4^		
	CON	TAL	TAM	TAH		Dose	L	Q
pH	6.81	6.99	6.91	6.84	0.074	0.344	0.645	0.320
NH_3_-N, mmol/L	13.0^a^	7.93^b^	10.1^b^	8.14^b^	0.902	<0.0001	0.006	0.146
TVFA, mmol/L	77.0^a^	74.4^a^	62.4^b^	81.0^a^	3.45	0.001	0.134	<0.0001
**VFA profile, mol/100 mol**								
Acetate	69.1^b^	69.7^b^	71.0^a^	71.4^a^	0.44	0.001	<0.0001	0.019
Propionate	16.1	16.4	15.5	15.9	0.33	0.201	0.473	0.111
Butyrate	11.3^a^	10.9^ab^	10.3^b^	10.3^b^	0.31	0.034	0.021	0.067
Isobutyrate	1.07^a^	0.89^b^	0.93^b^	0.65^c^	0.052	<0.0001	<0.0001	0.934
Valerate	1.53^a^	1.19^b^	1.26^b^	0.79^c^	0.079	<0.0001	<0.0001	0.679
Isovalerate	0.97	0.95	1.03	0.88	0.043	0.056	0.073	0.074
A:P	6.03^b^	6.29^b^	7.71^a^	6.07^b^	0.432	0.007	0.772	0.001

^1^TVFA, total volatile fatty acid; A:P, the ratio of acetate to propionate.

^2^CON, control group; TAL, low-dose tannic acid treatment, 0.3% DM; TAM, mid-dose tannic acid treatment, 0.9% DM; TAH, high-dose tannic acid treatment, 2.7% DM.

^3^SEM, standard error of means for treatments.

^4^L, linear effect of the tannic acid dose; Q, quadratic effect of the tannic acid dose.

^a,b^Means within a row for treatments (*P* < 0.05).

### Biochemical and physiological parameters of the blood serum

The level of INS in the serum of the cattle in the TAL treatment was significantly enhanced (*P* < 0.01) compared with the CON, whereas the quantity of CORT in the TAM group was significantly less (*P* < 0.01) than that in the TAL group. In addition, declines in the concentrations of MDA (linear, *P* < 0.05), T4 (linear, *P* < 0.01), and GC (quadratic, *P* < 0.01) to varying degrees as the supplemental level of TA rose were also observed (Table 4).

**TABLE 4:** Effects of tannic acid at different supplemental levels on biochemical and physiological parameters in the blood serum of Holstein bulls.

Items^1^	Treatments^2^				SEM^3^	*P*-value^4^		
	CON	TAL	TAM	TAH		Dose	L	Q
TP, g/L	79.4	82.9	81.1	78.6	2.86	0.963	0.958	0.638
ALB, g/L	34.6	36.5	34.6	33.8	1.58	0.632	0.378	0.830
IgA, g/L	0.58	0.65	0.60	0.55	0.048	0.440	0.286	0.499
IgG, g/L	4.65	4.50	5.53	4.75	0.526	0.484	0.834	0.187
CRE, μmol/L	88.6	99.6	82.0	84.2	13.52	0.787	0.575	0.774
GLU, mmol/L	3.55	3.92	3.88	3.99	0.186	0.268	0.167	0.391
TG, mmol/L	0.66	0.68	0.58	0.55	0.086	0.651	0.260	0.688
TC, mmol/L	4.26	4.18	4.05	3.74	0.367	0.701	0.239	0.938
HDLC, mmol/L	0.63	0.63	0.63	0.64	0.044	0.997	0.820	0.976
LDLC, mmol/L	4.06	3.81	4.17	4.04	0.462	0.954	0.881	0.850
MDA, nmol/L	11.2^a^	10.5^ab^	12.3^a^	7.6^b^	1.30	0.042	0.022	0.147
ALT, U/L	25.7	27.3	31.3	30.2	2.46	0.301	0.190	0.163
AST, U/L	61.1	52.1	51.6	61.0	4.06	0.144	0.400	0.052
GR, U/L	7.53	7.56	5.93	7.67	0.686	0.210	0.807	0.053
SOD, U/L	145	141	142	142	1.6	0.387	0.375	0.320
GH, ng/mL	6.92	5.76	3.16	8.34	1.928	0.183	0.319	0.054
T3, ng/mL	0.98	0.99	1.03	0.96	0.030	0.389	0.558	0.121
T4, ng/mL	45.4^a^	43.1^ab^	40.3^bc^	38.6^c^	1.21	0.001	<0.0001	0.052
CORT, μg/mL	0.24^ab^	0.34^a^	0.18^b^	0.34^a^	0.040	0.009	0.169	0.079
EPI, ng/mL	15.3	18.4	14.0	14.9	1.92	0.374	0.446	0.621
INS, ng/mL	1.09^bc^	1.57^a^	0.81^c^	1.30^ab^	0.148	0.004	0.817	0.067
GC, ng/mL	0.54^a^	0.46^ab^	0.40^b^	0.53^a^	0.056	0.040	0.575	0.005

^1^TP, total protein; ALB, albumin; IgA, immunoglobulin A; IgG, immunoglobulin G; CRE, creatinine; GLU, glucose; TG, triglyceride; TC, total cholesterol; HDLC, high-density lipoprotein cholesterol; LDLC, low-density lipoprotein cholesterol; MDA, malondialdehyde; ALT, alanine aminotransferase; AST, aspartate aminotransferase; GR, glutathione reductase; SOD, superoxide dismutase; GH, growth hormone; T3, triiodothyronine; T4, thyroxine; CORT, cortisol; EPI, epinephrine; INS, insulin; GC, glucagon.

^2^CON, control group; TAL, low-dose tannic acid treatment, 0.3% DM; TAM, mid-dose tannic acid treatment, 0.9% DM; TAH, high-dose tannic acid treatment, 2.7% DM.

^3^SEM, standard error of means for treatments.

^4^L, linear effect of the tannic acid dose; Q, quadratic effect of the tannic acid dose.

^a−c^Means within a row for treatments (*P* < 0.05).

### Lipopolysaccharide endotoxin and relevant inflammatory indicators

In this study, the levels of LPS in both rumen fluid and plasma, and concentrations of the related pro-inflammatory mediators including lipopolysaccharide binding protein (LBP), interleukin (IL)-6, and IL-8 in the cattle remained unchanged (*P* > 0.05) with the addition of TA (Table 5). However, supplementing TA lessened the amount of tumor necrosis factor alpha (TNF-α) (quadratic, *P* < 0.05) but increased the level of interferon gamma (IFN-γ) (quadratic, *P* < 0.01) when compared with the control, respectively. It is also noteworthy that the TNF-α concentration was significantly lower (*P* < 0.05) than that of the control.

**TABLE 5:** Effects of tannic acid at different supplemental levels on lipopolysaccharide endotoxin in the rumen liquid and plasma, and relevant inflammatory indicators in the serum of Holstein bulls.

Items^1^	Treatments^2^				SEM^3^	*P*-value^4^		
	CON	TAL	TAM	TAH		Dose	L	Q
LPS-R, EU/mL	14,900	16,653	16,176	14,929	731.1	0.169	0.403	0.124
LPS-P, EU/mL	11,433	11,905	11,112	12,089	343.5	0.172	0.231	0.238
LBP, ng/mL	18.6	27.1	29.2	25.2	5.05	0.434	0.616	0.174
IL-6, pg/mL	368	191	185	311	62.0	0.077	0.769	0.025
IL-8, pg/mL	358	440	156	372	92.5	0.142	0.927	0.071
TNF-α, ng/mL	8.77^a^	4.99^b^	4.70^b^	8.01^ab^	1.315	0.049	0.539	0.015
IFN-γ, pg/mL	245^b^	275^b^	567^a^	187^b^	94.6	0.019	0.430	0.004

^1^LPS-R, lipopolysaccharide in rumen liquid; LPS-P, lipopolysaccharide in plasma; LBP, lipopolysaccharide binding protein; IL-6, interleukin-6; IL-8, interleukin-8; TNF-α, tumor necrosis factor alpha; IFN-γ, interferon gamma.

^2^CON, control group; TAL, low-dose tannic acid treatment, 0.3% DM; TAM, mid-dose tannic acid treatment, 0.9% DM; TAH, high-dose tannic acid treatment, 2.7% DM.

^3^SEM, standard error of means for treatments.

^4^L, linear effect of the tannic acid dose; Q, quadratic effect of the tannic acid dose.

^a,b^Means within a row for treatments (*P* < 0.05).

### Taxonomic identification of ruminal bacteria

After the sequencing and following quality filtration of this study, an average of 11,876 ± 6,064 CCS clean reads and an average of 294 ± 146 OTUs per sample were obtained, respectively (Supplementary Table 1). Altogether, 19 bacterial phyla were annotated across the samples. Amongst them, *Proteobacteria* (71.9 ± 34.74%), *Firmicutes* (11.2 ± 15.15%), and *Bacteroidetes* (11.8 ± 14.20%) were identified as the top three predominant phyla and together occupied 94.8 ± 6.64% of the entire bacterial populations across all the samples (Supplementary Figure 1). At the genus level, a total of 94 bacterial genera were observed from all the samples, with *Ralstonia* (68.3 ± 39.64%), *Bradyrhizobium* (2.5 ± 9.98%), and *Succiniclasticum* (1.9 ± 2.93%) generally being the most dominant and a joint proportion within the bacterial community at 72.7 ± 34.11% (Supplementary Figure 2). In total, 91 species were detected amongst all the rumen fluid samples of this study, and the ruminal bacterial microbiome at the species level were basically predominated by the *Bradyrhizobium* sp. (2.5 ± 9.90%), *Prevotella ruminicola* (0.4 ± 0.77%), SR1 bacterium human oral taxon HOT-345 (0.4 ± 0.86%), *Lachnospiraceae* bacterium CA43 (0.1 ± 0.32%), and *Ralstonia pickettii* (0.4 ± 0.38%) (Supplementary Figure 3). In all, 786, 842, 1,060, and 1,029 OTUs were clustered in the CON, TAL, TAM, and TAH, from which 123, 109, 200, and 227 were found as exclusive to each treatment, respectively (Supplementary Figure 4). In general, the overwhelming majority of those unique OTUs in each group was annotated to the phyla of either *Firmicutes*, *Bacteroidetes*, *Proteobacteria*, or *Tenericutes*.

### Diversity of rumen bacterial community

**FIGURE 1:**
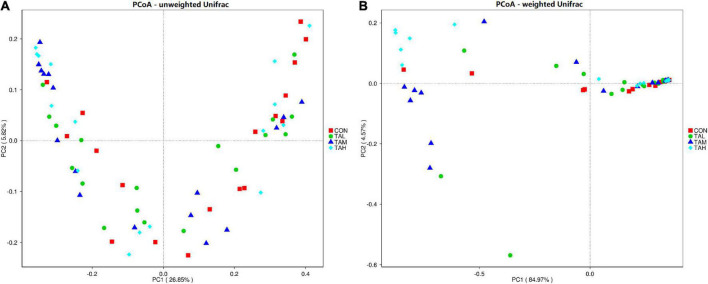
The principal coordinate analysis (PCoA) plots on rumen bacterial community structure across the four treatments. **(A)** PCoA based on the unweighted Unifrac matrix. **(B)** PCoA based on the weighted Unifrac matrix.

As to the Alpha diversity of rumen bacterial microbiome, the dose of TA significantly influenced (*P* < 0.05) the indices of Shannon and Simpson, with a highly significant (*P* < 0.01) and a significant (*P* < 0.05) linear increment noticed, respectively (Table 6). The Shannon index of the rumen bacterial populations in TAH and the Simpson index in TAM were significantly elevated (*P* < 0.05) compared with the CON. For the Beta diversity of the bacterial microbiota in the rumen liquid across different treatments, the PCoA analysis was calculated and visualized using the unweighted and weighted Unifrac matrix, respectively (Figure 1). There was no obvious distinction within the bacterial communities across different groups, as the treatment-dependent clustering was exhibited in neither of the two plots.

**TABLE 6:** Effects of tannic acid at different supplemental levels on Alpha diversity indices of the bacterial community in the rumen fluid of Holstein bulls.

Items	Treatments^1^				SEM^2^	*P*-value^3^		
	CON	TAL	TAM	TAH		Dose	L	Q
Ace	233	300	324	342	38.6	0.178	0.088	0.246
Chao 1	192	246	274	301	35.5	0.142	0.048	0.300
Shannon	1.61^b^	1.93^ab^	3.52^ab^	3.55^a^	0.533	0.012	0.008	0.065
Simpson	0.25^b^	0.31^ab^	0.51^a^	0.49^ab^	0.073	0.031	0.025	0.060

^1^CON, control group; TAL, low-dose tannic acid treatment, 0.3% DM; TAM, mid-dose tannic acid treatment, 0.9% DM; TAH, high-dose tannic acid treatment, 2.7% DM.

^2^SEM, standard error of means for treatments.

^3^L, linear effect of the tannic acid dose; Q, quadratic effect of the tannic acid dose.

^a,b^Means within a row for treatments (*P* < 0.05).

### Differential rumen bacterial taxa within treatments

**FIGURE 2:**
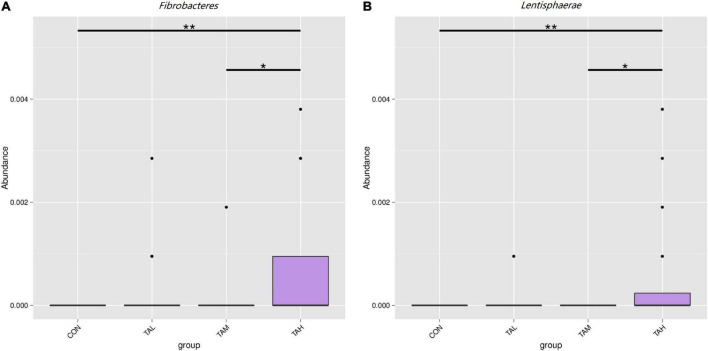
Differential bacterial phyla in the rumen fluid across the four treatments. **(A)** *Fibrobacteres*. **(B)** *Lentisphaerae*. *Means that the difference between two treatments was significant (*P* < 0.05). **Means that the difference between two treatments was highly significant (*P* < 0.01).

**FIGURE 3:**
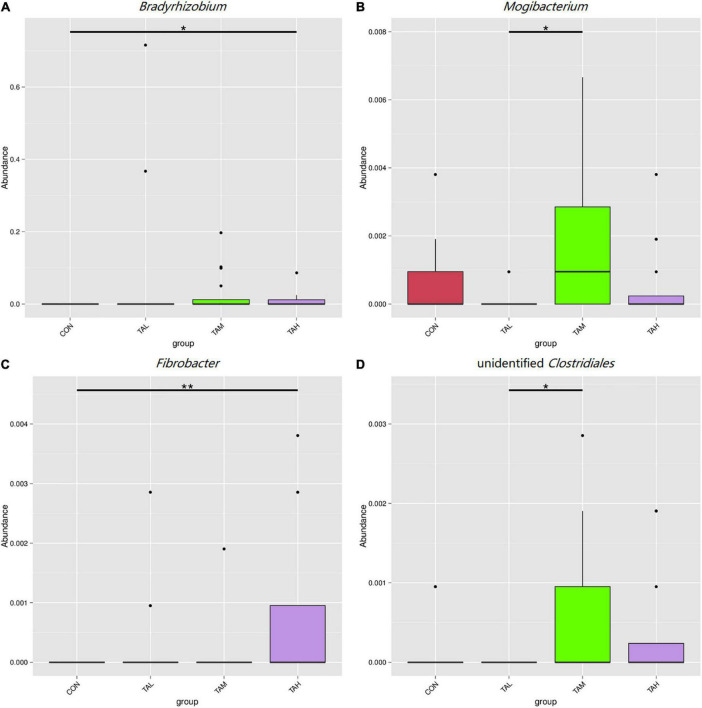
Differential bacterial genera in the rumen fluid across the four treatments. **(A)** *Bradyrhizobium*. **(B)** *Mogibacterium*. **(C)** *Fibrobacter*. **(D)** Unidentified *Clostridiales*. *Means that the difference between two treatments was significant (*P* < 0.05). **Means that the difference between two treatments was highly significant (*P* < 0.01).

**FIGURE 4:**
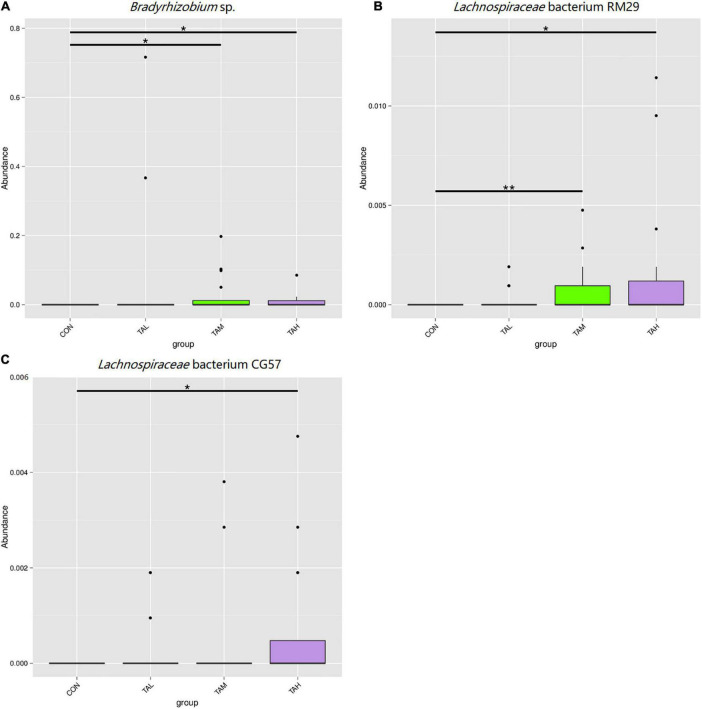
Differential bacterial species in the rumen fluid across the four treatments. **(A)** *Bradyrhizobium* sp. **(B)** *Lachnospiraceae* bacterium RM29. **(C)** *Lachnospiraceae* bacterium CG57. *Means that the difference between two treatments was significant (*P* < 0.05). **Means that the difference between two treatments was highly significant (*P* < 0.01).

As was illustrated by the Metastats analysis, the relative proportions of the phyla *Fibrobacteres* and *Lentisphaerae* in the rumen fluid of the cattle from the TAH treatment were highly significantly elevated (*q* < 0.01) compared with the CON and TAL, and significantly higher (*q* < 0.05) than those in the TAM, respectively (Figure 2). For the differential taxa at the genus level, it was observed that adding TA at 2.7% DM significantly (*q* < 0.05) and highly significantly (*q* < 0.01) raised the abundances of *Bradyrhizobium* and *Fibrobacter* in the rumen liquid when compared to the CON, respectively (Figure 3). Besides, a significant exceedance (*q* < 0.05) in the abundances of both *Mogibacterium* and unidentified *Clostridiales* in the TAM relative to the TAL was also noticed. As to the distinct bacterial species, the relative proportions of *Bradyrhizobium* sp., *Lachnospiraceae* bacterium RM29, and *Lachnospiraceae* bacterium CG57 were significantly increased (*q* < 0.05) in response to the TA inclusion at 2.7% DM (Figure 4). Moreover, a significant advantage (*q* < 0.05) and a highly significant preponderance (*q* < 0.01) in the relative proportions of *Bradyrhizobium* sp. and *Lachnospiraceae* bacterium RM29 in the TAM compared to TAL were separately demonstrated.

### Function prediction of rumen bacterial microbiome

**FIGURE 5:**
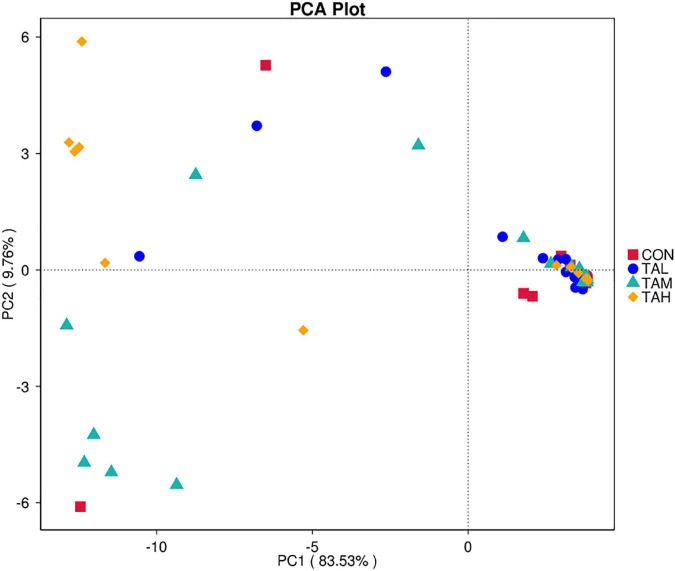
The principle component analysis (PCA) plot for the predicted metagenome across the four treatments based on Tax4Fun.

In total, 7.4 ± 9.59% of OTUs mapped to the SILVA database were assigned to KEGG orthologs (KO) and relevant pathways (at level 2) through the function prediction via Tax4Fun. The top 10 KEGG pathways across treatments revealed by Tax4Fun were depicted in Supplementary Figure 5, amongst which the carbohydrate metabolism, membrane transport, replication and repair, translation, and amino acid metabolism were primarily detected with the most predominant KO abundances. As was revealed by the principle component analysis (PCA) diagram, there was no clear separation in the assigned KEGG pathways within different treatments (Figure 5). As demonstrated by the *t*-test analysis, there was no significant (*P* > 0.05) difference in the KO abundances of the most dominant KEGG pathways within treatments (Supplementary Table 2).

## Discussion

Due to the lowered palatability, decreased evacuation rate of digesta out of rumen, and toxicity, tannins could exert negative effects on the feed intake of ruminants (37). Nevertheless, several precedent studies conducted in ruminants have found that the feed intake was unaffected by the supplementation of either CTs or HTs in relatively small amounts. Henke et al. (13) showed no effect on the intake of DM and OM by adding CT (quebracho extract) at either 1.5% or 3.0% DM in the ration of Holstein dairy cows. Moreover, Aboagye et al. (14) found that neither the sole inclusion of HT from chestnut extract (0.25% or 1.5% DM), nor the even combination of chestnut HT and quebracho CT (0.125% or 0.75% DM of each) affected the DMI of beef cattle fed a high-forage diet. Further, it was revealed that none of the supplementation of bayberry CT, *Acacia mangium* CT, and valonia HT at 3% DM significantly alter the nutrients intake of lactating dairy cows (10). In the current study, however, adding TA at 2.7% DM significantly reduced the intake of DM and OM, preliminarily suggesting that the TA (extracted from *Rhus chinensis* Mill.) used in this trial could possess a depressed palatability for the Holstein bulls compared with the CTs and HTs in previous investigations.

Unlike the effects on feed intake, previous studies have displayed the negative impacts of CTs or HTs even supplemented at small proportions on the nutrient digestibility of ruminants to different extents. It was demonstrated that supplementing 3% *Acacia mangium* CT decreased the apparent total tract digestibility of DM, NDF, ADF, and CP, whilst the addition of 3% valonia HT only lowered the CP digestibility of dairy cattle (10). Yang et al. (5) has reported that adding TA at 0.65, 1.3, and 2.6% DM reduced the CP digestibility, and the addition of TA at 2.6% DM decreased the digestibility of DM and OM in beef cattle. Zhou et al. (15) also noticed the reduction in the digestibility of DM, OM, and CP of beef cattle by the inclusion of 1.69% TA. Similarly, in the present study, the dietary supplementation of TA at 0.9 and 2.7% DM both decreased the apparent digestibility of DM, OM, and CP of the Holstein bulls. The declines in the nutrients digestibility might result from the binding effects of tannins to proteins, minerals and polysaccharides (e.g., cellulose, hemicellulose, pectin, and starch) in the diet of ruminants, and the incomplete disintegration of these mixtures in the abomasum (38, 39).

In this trial, the ADG and the ratio of gain to feed (G:F) remained unaffected by the supplementation of TA at three different doses. This result was in line with the report of Aboagye et al. (14), in which no effects of either the single addition of HT or the evenly combined inclusion of HT and CT on the ADG and G:F of beef cattle were exhibited. Likewise, it was also found that supplementing neither 1.0% nor 3.0% chestnut HT changed the ADG and feed conversion ratio of sheep (4). Due to the binding ability of tannins to proteins, the quantity of RUP could be increased, and the flux of MP into the lower digestive tract might possibly be raised (2). Since the increment of MP flow into the small intestine could enhance the absorption of amino acids into the blood and thereby contribute to the improvement of growth performance, it could assumed that the amount of MP was not elevated by adding TA at different levels (11), or the nutrients provided by the basal diet was already sufficient for the growth requirement of Holstein bulls in this study (14).

When TA was added in the diet of cattle at different doses in the present experiment, the ruminal concentration of NH_3_-N was reduced significantly. Likewise, significant declines of the NH_3_-N level in rumen fluid in responses to the TA supplementation at different levels have been marked in previous studies (2, 5). This result could be explained by the ability of TA to bind to dietary CP and hence the suppression of CP degradation by the ruminal microorganisms (40). Moreover, adding 0.9% TA lowered the ruminal TVFA concentration, and the three supplemental levels of TA decreased the molar percentages of butyrate, isobutyrate, and valerate in different manners. Zhou et al. (15) also found the decreases in the TVFA concentration, as well as the molar ratios of valerate and isovalerate in the rumen liquid of beef cattle by the inclusion of TA at 1.69% DM. The reductions in the concentrations of ruminal VFAs imply the inhibition of dietary carbohydrates fermentation by TA, which could be attributed to the binding capacity of TA with carbohydrates, or the suppressive effects of TA on some specific ruminal microbes. However, as was illustrated in the current study and precedent report (2), the apparent digestibility of NDF and ADF both remained unaffected in response to the TA supplementation. Therefore, it could be presumed that TA probably mainly hinder the ruminal degradation of non-structural carbohydrates but requires further investigations. Further, considering that there was no significant difference between the apparent nutrient digestibilities, the increment of TVFA in TAH compared with TAM might result from the possibly decreased VFA clearance rate in the rumen. In addition, the significant increments in the acetate proportion and acetate to propionate ratio in the TAM group were also noticed, which is also in line with the study on the addition of 1.69% TA of Zhou et al. (15).

As a biomarker of the lipid peroxidation, MDA is commonly measured to reflect the status of oxidative stress in the ruminants (41, 42). In this study, the serum MDA level of Holstein cattle was significantly reduced in TAH compared to the CON, indicating that the oxidative injury was relieved by supplementing 2.7% TA. This result is similar to the finding of Ugur Calis et al. (18) and further indicates that the antioxidant activity of TA might rely on its dosage, whereas the detailed antioxidative mechanisms in animals requires further investigations to be revealed. The circulating concentration of T4 in ruminants is bound up with the thyroid function, and susceptible to the variations in environment and physiological conditions (43). The significant decline in the T4 level in TAH suggested a potential suppression of the thyroid function by the inclusion of TA at 2.7% DM in the present experiment (44). Moreover, T4 has been used as an oxidative stress inducer in rats (45), and it was found that the administration of T4 in drinking water at 2 mg/L significantly raised the concentration of MDA in the cardiac muscle of rats (46). Therefore, the reduction of T4 in TAH could be a cause for the decrease in MDA. In addition, we observed the significant reduction of the circulating GC concentration in the cattle of TAM treatment compared with the CON. As a typical mechanism involving gluconeogenesis of ruminants, the secretion of glucagon can be potently activated by the ruminally derived VFA, especially the propionate and butyrate (47–49). For this reason, the decrease in GC level in the TAM treatment might result from the above-mentioned diminishing of ruminal TVFA concentration and butyrate proportion in the same group of this trial.

The LPS endotoxin released by the gram-negative bacteria can stimulate the production of several pro-inflammatory cytokines and thereby trigger systemic inflammation, when it translocates into the peripheral blood from the digestive tract (50, 51). As demonstrated in the current experiment, the density of LPS neither in the rumen fluid nor the plasma was influenced by the addition of TA at different doses. Nevertheless, the concentration of TNF-α, a cytokine induced by LPS (52), was lowered by supplementing TA at both 0.3 and 0.9% DM, indicating a possible mitigation of the inflammatory responses of the bulls. Besides, the rise in the level of IFN-γ, also a LPS-relevant cytokine (53), was exhibited in the TAM treatment. The alterations of these cytokines in response to the TA inclusion necessitates future studies to be explained.

The present experiment showed the successive predominance of the three phyla *Proteobacteria*, *Firmicutes*, and *Bacteroidetes* in the rumen bacterial microflora of the Holstein bulls, supporting the similar findings of precedent investigations, including our previous reports on the dominant ruminal bacterial phyla in ruminants (19, 32, 34, 54, 55). Besides, the dominance of the genus *Ralstonia*, of which the species *Ralstonia pickettii* has been previously found in the rumen of dairy cows (56), was also observed. This phenomenon might result from the conditions of environment, diet, and cattle of the present trial, and requires further investigations to be explained. In addition, at each bacterial taxonomic level, evident individual variations in the relative abundance were noticed in this trial. The various sources of the bacterial colonization and their interactions, as well as the host genetics could account for the individual variations (57). It was found that despite the substantial differences in the composition of rumen bacterial populations, Holstein cows fed identical diet still presented similar rumen fermentation characteristics, milk yield, and milk composition (58). Likewise, the ADG and G:F of the cattle were similar regardless of the individual variations and distinctions across different treatments in the ruminal bacterial microbiome of this study. The diversity and richness of ruminal microbiota are key elements that influence the rumen functionality (59). Zhou et al. (15) noticed that the Shannon index of rumen bacterial community in beef cattle was raised by supplementing 1.69% TA. In the current trial, the addition of 2.7% TA elevated the Shannon index, while the Simpson index in the TAM group was higher than that of the CON, suggesting the differences in the impacts on the bacterial diversity at different doses. Further, as depicted by both the PCoA diagram based on both unweighted and weighted Unifrac matrix, supplementing TA at different levels did not alter the bacterial community structure in the rumen fluid of the bulls. The correlations between the responses of bacterial diversity and rumen fermentation in response to the TA addition at different levels in this study requires further research to be elucidated.

The phylum *Fibrobacteres* that consists of one formal genus *Fibrobacter*, is eminently competent in degrading lignocellulose into acetate and succinate, mainly in the mammalian gastrointestinal tract (60, 61). As demonstrated in this study, the abundances of both *Fibrobacteres* and *Fibrobacter* are highly significantly increased in the rumen fluid of cattle in the TAH treatment than the CON group, potentially contributing to the above-described escalation of the ruminal acetate ratio. Besides, a highly significant increment in the proportion of the phylum *Lentisphaerae* when TA was added at 2.7% was also marked. With a common relative proportion below 1% amid the ruminal microflora, *Lentisphaerae* has found to be correlated with the feed efficiency of ruminants, but its exact role in feed efficiency still remains uncertain (62–64). Here in this trial, the gain to feed ratio in TAH was not changed along with the elevated abundance of *Lentisphaerae*, necessitating deeper explorations to be interpreted. As typical nitrogen-fixing bacteria that colonizes the root of many sorts of plants (65), *Bradyrhizobium* spp. were previously detected with very low prevalence in the rumen of impala (66). The presence and increase in the proportions of *Bradyrhizobium* and *Bradyrhizobium* sp. in response to TA supplementation of the current trial needs to be elucidated. Gagen et al. (67) had verified that *Lachnospiraceae* is capable of utilizing hydrogen and carbon dioxide to produce acetate, which could act as an efficient alternative to the ruminal methanogenesis. Therefore, the growth in the abundances of *Lachnospiraceae* bacterium RM29 in both TAM and TAH, and *Lachnospiraceae* bacterium CG57 in TAH might partially lead to the increases of ruminal acetate proportion in TAM and TAH of this study. Further, based on the methane-reducing effects of tannins reported previously (4, 5, 14), it could be inferred that the variations of those two bacterial species belonging to *Lachnospiraceae* might be linked to the probable suppressed methanogeneis in the present trial. Moreover, the discrepancy between the general unchangeableness in the KEGG pathways and the alterations in rumen fermentation parameters within different treatments of this study could stem from the limits of Tax4Fun analysis, differences between the liquid- and solid-phase bacteria, and disparity between metagenomics analysis and actual metabolisms of the rumen microbiome (19).

## Conclusion

In the present study, supplementing TA at 0.3% DM alleviated the inflammatory responses and tended to raise G:F, without impairing the nutrient intake, ADG, apparent nutrient digestibility, and ruminal TVFA concentration of Holstein bulls. By contrast, the inclusion of TA at 0.9 and 2.7% DM reduced either the nutrient intake, nutrient digestibility, or the TVFA level, despite that the cattle performance was also unaffected. As for the bacterial microbiome in rumen fluid, significant impacts on the Shannon index, and the abundances of several bacteria at different taxonomic levels of the 2.7% TA addition were demonstrated. As a consequence, it is suggested that the 0.3% TA supplementation would be more preferred compared with other doses, when the cattle performance and the TA cost are considered comprehensively.

## Data availability statement

The datasets presented in this study can be found in online repositories. The names of the repository/repositories and accession number(s) can be found below: https://www.ncbi.nlm.nih.gov/, PRJNA770103.

## Ethics statement

This animal study was reviewed and approved by the Animal Care Committee, College of Animal Science and Technology, Hunan Agricultural University.

## Author contributions

ZW, FW, and WS designed the research. ZW, YZ, XL, JH, FW, WS, ST, CZ, ZT, and YY conducted the research. ZW and YZ analyzed the data. ZW wrote the manuscript. All authors approved the final manuscript.
