# Supplementary material for: Tannic acid supplementation in the diet of Holstein bulls: Impacts on production performance, physiological and immunological characteristics, and ruminal microbiota
Source: Front Nutr. 2022 Nov 16;9:1066074. doi: 10.3389/fnut.2022.1066074 (PMC9709124; doi:10.3389/fnut.2022.1066074)
Supplement: Supplementary file 1 [file Image_1.pdf]

Supplementary Material

Tannic acid supplementation in the diet of Holstein bulls: Impacts on production performance, physiological and immunological characteristics, and ruminal microbiota

Zuo Wang [1], Yuan Zhao [1], Xinyi Lan [1], Jianhua He [1], Fachun Wan [1]*, Weijun Shen [1]*, Shaoxun Tang [2], Chuanshe Zhou [2], Zhiliang Tan [2], Yanming Yang [3]

[1] College of Animal Science and Technology, Hunan Agricultural University, Changsha, Hunan 410128, China

[2] CAS Key Laboratory of Agro-Ecological Processes in Subtropical Region, National Engineering Laboratory for Pollution Control and Waste Utilization in Livestock and Poultry Production, Hunan Provincial Key Laboratory of Animal Nutrition & Physiology and Metabolism, Institute of Subtropical Agriculture, Chinese Academy of Sciences, Changsha, Hunan 410125, China

[3] Jiurui Biology & Chemistry Co. Ltd., Zhangjiajie, Hunan 427000, China

*** Correspondence:**

Fachun Wan; Weijun Shen

wanfc@sina.com; shenweijun@hunau.edu.cn

Supplementary Figures

Figure S1. Relative abundances of top 10 bacterial phylum

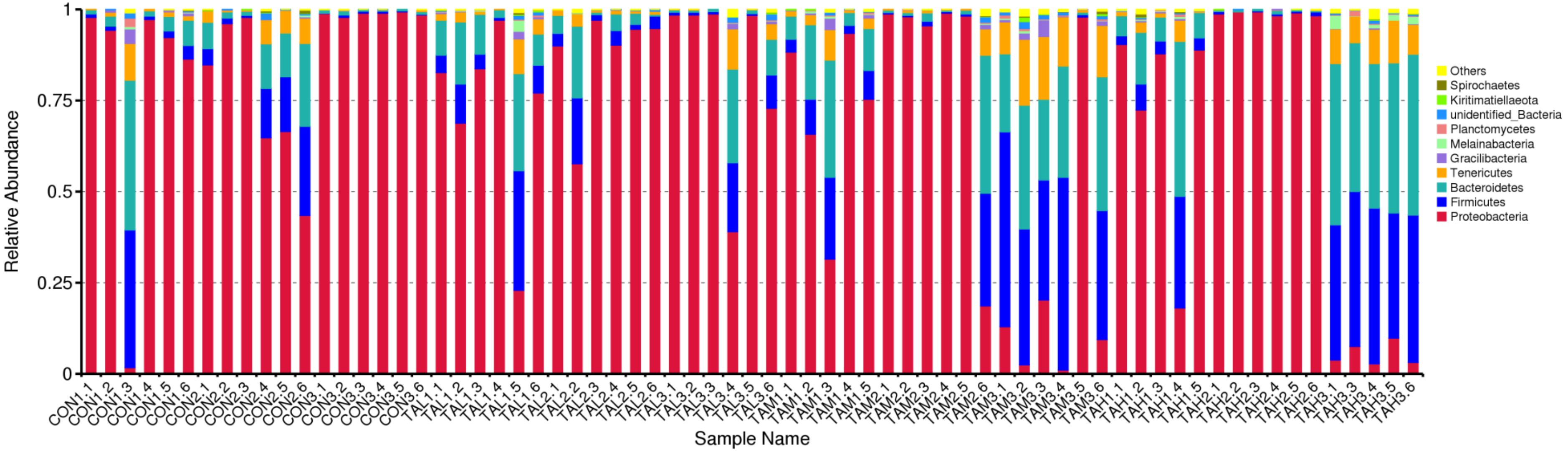

Relative Abundance
1
0.75
0.5
0.25
0
Sample Name
CON1.1 CON1.2 CON1.3 CON1.4 CON1.5 CON1.6 CON2.1 CON2.2 CON2.3 CON2.4 CON2.5 CON2.6 CON3.1 CON3.2 CON3.3 CON3.4 CON3.5 CON3.6
TAL1.1 TAL1.2 TAL1.3 TAL1.4 TAL1.5 TAL1.6 TAL2.1 TAL2.2 TAL2.3 TAL2.4 TAL2.5 TAL2.6 TAL3.1 TAL3.2 TAL3.3 TAL3.4 TAL3.5 TAL3.6
TAM1.1 TAM1.2 TAM1.3 TAM1.4 TAM1.5 TAM2.1 TAM2.2 TAM2.3 TAM2.4 TAM2.5 TAM2.6 TAM3.1 TAM3.2 TAM3.3 TAM3.4 TAM3.5 TAM3.6
TAH1.1 TAH1.2 TAH1.3 TAH1.4 TAH1.5 TAH2.1 TAH2.2 TAH2.3 TAH2.4 TAH2.5 TAH2.6 TAH3.1 TAH3.3 TAH3.4 TAH3.5 TAH3.6
Others
Spirochaetes
Kiritimatiellaeota
unidentified_Bacteria
Planctomycetes
Melainabacteria
Gracilibacteria
Tenericutes
Bacteroidetes
Firmicutes
Proteobacteria
