# Supplementary material for: Tannic acid supplementation in the diet of Holstein bulls: Impacts on production performance, physiological and immunological characteristics, and ruminal microbiota
Source: Front Nutr. 2022 Nov 16;9:1066074. doi: 10.3389/fnut.2022.1066074 (PMC9709124; doi:10.3389/fnut.2022.1066074)
Supplement: Supplementary file 2 [file Image_2.pdf]

Supplementary Material

Tannic acid supplementation in the diet of Holstein bulls: Impacts on production performance, physiological and immunological characteristics, and ruminal microbiota

Zuo Wang [1], Yuan Zhao [1], Xinyi Lan [1], Jianhua He [1], Fachun Wan [1]*, Weijun Shen [1]*, Shaoxun Tang [2], Chuanshe Zhou [2], Zhiliang Tan [2], Yanming Yang [3]

[1] College of Animal Science and Technology, Hunan Agricultural University, Changsha, Hunan 410128, China

[2] CAS Key Laboratory of Agro-Ecological Processes in Subtropical Region, National Engineering Laboratory for Pollution Control and Waste Utilization in Livestock and Poultry Production, Hunan Provincial Key Laboratory of Animal Nutrition & Physiology and Metabolism, Institute of Subtropical Agriculture, Chinese Academy of Sciences, Changsha, Hunan 410125, China

[3] Jiurui Biology & Chemistry Co. Ltd., Zhangjiajie, Hunan 427000, China

*** Correspondence:**

Fachun Wan; Weijun Shen

wanfc@sina.com; shenweijun@hunau.edu.cn

Supplementary Figures

Figure S2. Relative abundances of top 10 bacterial genus

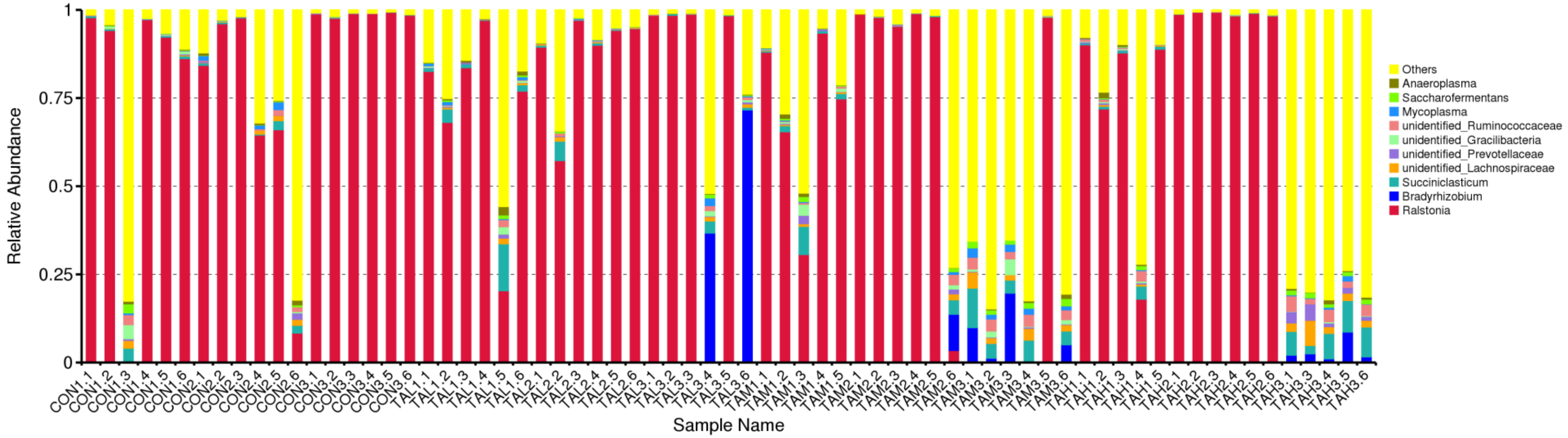
Relative Abundance
1
0.75
0.5
0.25
0
CON1.1
CON1.2
CON1.3
CON1.4
CON1.5
CON1.6
CON2.1
CON2.2
CON2.3
CON2.4
CON2.5
CON2.6
CON3.1
CON3.2
CON3.3
CON3.4
CON3.5
CON3.6
TAL1.1
TAL1.2
TAL1.3
TAL1.4
TAL1.5
TAL1.6
TAL2.1
TAL2.2
TAL2.3
TAL2.4
TAL2.5
TAL2.6
TAL3.1
TAL3.2
TAL3.3
TAL3.4
TAL3.5
TAL3.6
TAM1.1
TAM1.2
TAM1.3
TAM1.4
TAM1.5
TAM2.1
TAM2.2
TAM2.3
TAM2.4
TAM2.5
TAM2.6
TAM3.1
TAM3.2
TAM3.3
TAM3.4
TAM3.5
TAM3.6
TAH1.1
TAH1.2
TAH1.3
TAH1.4
TAH1.5
TAH2.1
TAH2.2
TAH2.3
TAH2.4
TAH2.5
TAH2.6
TAH3.1
TAH3.3
TAH3.4
TAH3.5
TAH3.6
Sample Name
Others
Anaeroplasma
Saccharofermentans
Mycoplasma
unidentified_Ruminococcaceae
unidentified_Gracilibacteria
unidentified_Prevotellaceae
unidentified_Lachnospiraceae
Succiniclasticum
Bradyrhizobium
Ralstonia
