# Supplementary material for: Tannic acid supplementation in the diet of Holstein bulls: Impacts on production performance, physiological and immunological characteristics, and ruminal microbiota
Source: Front Nutr. 2022 Nov 16;9:1066074. doi: 10.3389/fnut.2022.1066074 (PMC9709124; doi:10.3389/fnut.2022.1066074)
Supplement: Supplementary file 5 [file Image_5.pdf]

Supplementary Material

Tannic acid supplementation in the diet of Holstein bulls: Impacts on production performance, physiological and immunological characteristics, and ruminal microbiota

Zuo Wang [1], Yuan Zhao [1], Xinyi Lan [1], Jianhua He [1], Fachun Wan [1]*, Weijun Shen [1]*, Shaoxun Tang [2], Chuanshe Zhou [2], Zhiliang Tan [2], Yanming Yang [3]

[1] College of Animal Science and Technology, Hunan Agricultural University, Changsha, Hunan 410128, China

[2] CAS Key Laboratory of Agro-Ecological Processes in Subtropical Region, National Engineering Laboratory for Pollution Control and Waste Utilization in Livestock and Poultry Production, Hunan Provincial Key Laboratory of Animal Nutrition & Physiology and Metabolism, Institute of Subtropical Agriculture, Chinese Academy of Sciences, Changsha, Hunan 410125, China

[3] Jiurui Biology & Chemistry Co. Ltd., Zhangjiajie, Hunan 427000, China

*** Correspondence:**

Fachun Wan; Weijun Shen

wanfc@sina.com; shenweijun@hunau.edu.cn

Supplementary Figures

Figure S5. The top 10 assigned KEGG pathways (at level 2) across the four treatments based on Tax4Fun

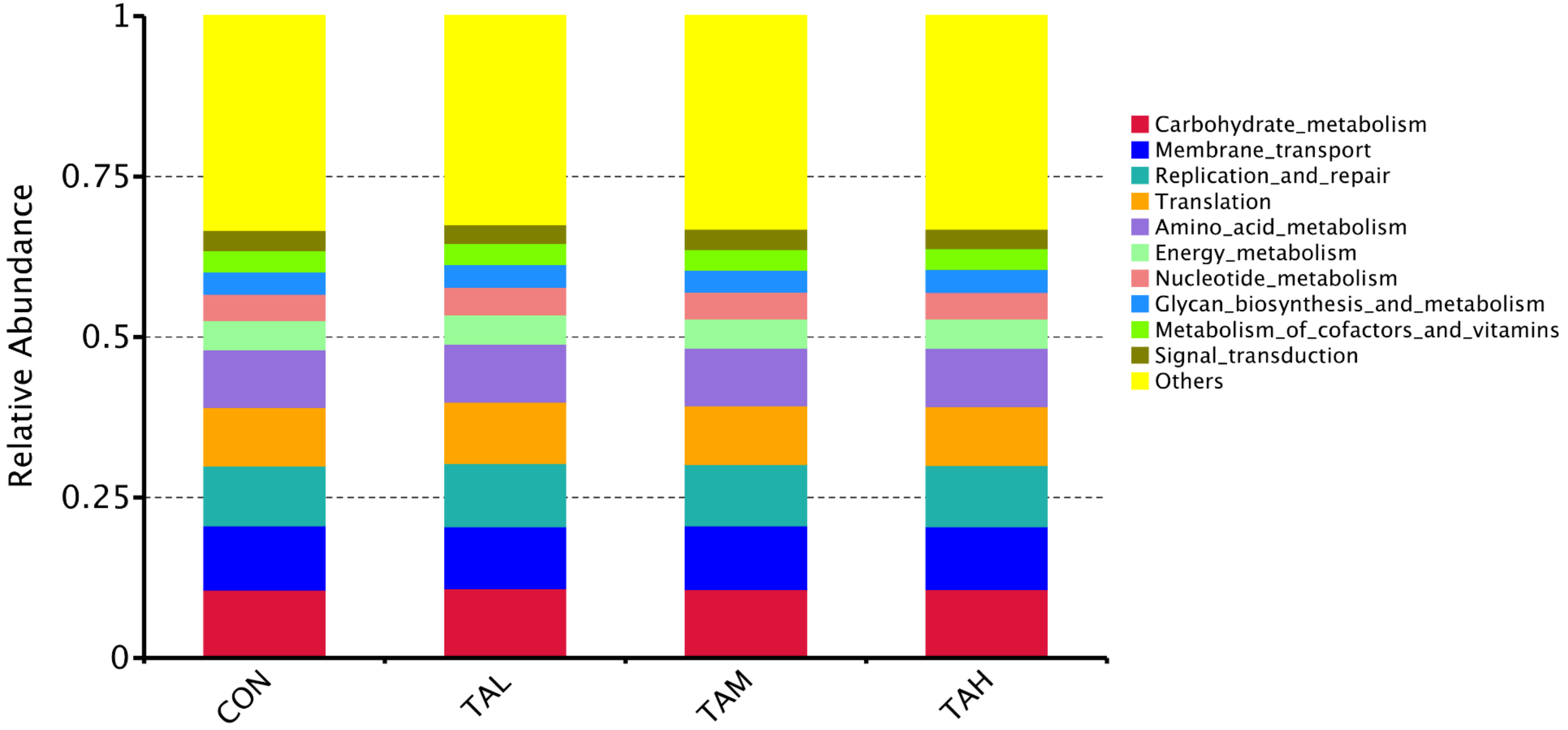
Relative Abundance
1
0.75
0.5
0.25
0
CON
TAL
TAM
TAH
Carbohydrate_metabolism
Membrane_transport
Replication_and_repair
Translation
Amino_acid_metabolism
Energy_metabolism
Nucleotide_metabolism
Glycan_biosynthesis_and_metabolism
Metabolism_of_cofactors_and_vitamins
Signal_transduction
Others
