# Supplementary material for: Tannic acid supplementation in the diet of Holstein bulls: Impacts on production performance, physiological and immunological characteristics, and ruminal microbiota
Source: Front Nutr. 2022 Nov 16;9:1066074. doi: 10.3389/fnut.2022.1066074 (PMC9709124; doi:10.3389/fnut.2022.1066074)
Supplement: Supplementary file 6 [file Table_1.PDF]

Supplementary Material

Tannic acid supplementation in the diet of Holstein bulls: Impacts on production performance, physiological and immunological characteristics, and ruminal microbiota

Zuo Wang [1], Yuan Zhao [1], Xinyi Lan [1], Jianhua He [1], Fachun Wan [1]*, Weijun Shen [1]*, Shaoxun Tang [2], Chuanshe Zhou [2], Zhiliang Tan [2], Yanming Yang [3]

[1] College of Animal Science and Technology, Hunan Agricultural University, Changsha, Hunan 410128, China

[2] CAS Key Laboratory of Agro-Ecological Processes in Subtropical Region, National Engineering Laboratory for Pollution Control and Waste Utilization in Livestock and Poultry Production, Hunan Provincial Key Laboratory of Animal Nutrition & Physiology and Metabolism, Institute of Subtropical Agriculture, Chinese Academy of Sciences, Changsha, Hunan 410125, China

[3] Jiurui Biology & Chemistry Co. Ltd., Zhangjiajie, Hunan 427000, China

*** Correspondence:**

Fachun Wan; Weijun Shen

wanfc@sina.com; shenweijun@hunau.edu.cn

Supplementary Tables

Table S1. Summary of sequencing data

Sample name	Raw reads(#)	Clean reads(#)	Base(nt)	AvgLen(nt)	Effective%	OTU num	Observed otus	shannon	simpson	chao1	ace	Goods coverage	PD whole tree
CON1.1	16983	14437	21105199	1461	85.01	177	22	0.379	0.073	31.429	44.511	0.989	6.376
CON1.2	10462	9327	13627736	1461	89.15	221	47	0.742	0.137	122.429	130.619	0.969	12.212
CON1.3	8378	7313	10685092	1461	87.29	463	358	7.734	0.991	507.188	530.239	0.853	44.724
CON1.4	13852	11927	17431454	1461	86.1	168	32	0.421	0.076	149	214.064	0.974	8.857
CON1.5	11150	9752	14256559	1461	87.46	308	73	1.01	0.179	213	271.344	0.947	17.121
CON1.6	9932	8278	12091567	1460	83.35	310	100	1.578	0.281	219.895	266.211	0.935	21.485
CON2.1	10436	8564	12504597	1460	82.06	330	109	1.706	0.305	267.053	345.953	0.926	21.524
CON2.2	16823	14941	21838118	1461	88.81	254	36	0.514	0.095	111.6	148.794	0.973	10.94
CON2.3	17034	14752	21566260	1461	86.6	200	24	0.368	0.069	48	66.507	0.985	7.546
CON2.4	10780	9093	13282330	1460	84.35	472	225	3.664	0.596	508.125	537.347	0.856	33.238
CON2.5	6757	5423	7934976	1463	80.26	323	174	3.291	0.573	304	380.919	0.9	26.64
CON2.6	10064	7279	10632480	1460	72.33	366	254	5.938	0.936	383	426.473	0.876	36.129
CON3.1	22314	18272	26707645	1461	81.89	132	15	0.227	0.043	42.5	55.944	0.99	3.992
CON3.2	12269	10730	15688625	1462	87.46	150	31	0.386	0.069	206.5	245.2	0.974	8.977
CON3.3	13184	11439	16724761	1462	86.76	92	15	0.241	0.047	70	62.012	0.99	5.113
CON3.4	25004	20077	29351100	1461	80.3	128	14	0.195	0.038	80	147.184	0.989	5.236
CON3.5	30871	26499	38741944	1462	85.84	121	9	0.127	0.025	30	53.722	0.993	3.726
CON3.6	28925	25268	36935979	1461	87.36	200	19	0.259	0.049	79	179.922	0.985	5.882
TAL1.1	9020	7969	11638203	1460	88.35	345	125	1.948	0.339	267.8	307.755	0.919	23.195
TAL1.2	11468	9278	13543691	1459	80.9	424	195	3.276	0.556	396.366	505.35	0.877	31.72
TAL1.3	9568	8475	12378169	1460	88.58	288	107	1.776	0.319	212	259.646	0.933	18.673
TAL1.4	13751	11392	16648058	1461	82.84	175	24	0.423	0.082	45	55.954	0.986	7.21
TAL1.5	7847	6774	9895412	1460	86.33	458	324	6.638	0.946	471.542	520.651	0.851	42.695
TAL1.6	8884	7715	11272621	1461	86.84	352	159	2.507	0.429	348.033	363.75	0.898	28.22

TAL2.1	9739	7893	11533789	1461	81.05	278	86	1.268	0.227	281	341.443	0.937	18.081
TAL2.2	6692	5313	7766272	1461	79.39	269	187	4.029	0.683	261.592	281.269	0.918	28.629
TAL2.3	19158	15913	23262901	1461	83.06	244	33	0.397	0.071	250.5	503.473	0.971	10.009
TAL2.4	10424	9244	13509982	1461	88.68	309	88	1.21	0.212	245.929	268.776	0.936	18.383
TAL2.5	9096	7327	10709197	1461	80.55	177	52	0.736	0.132	130.111	140.735	0.964	13.642
TAL2.6	12929	11089	16228146	1463	85.77	215	48	0.659	0.12	135.875	266.511	0.964	11.148
TAL3.1	18909	16886	24684736	1461	89.3	162	20	0.264	0.049	156	156.076	0.984	6.234
TAL3.2	20253	17332	25342849	1462	85.58	163	18	0.273	0.052	63.5	95.148	0.987	5.373
TAL3.3	20879	17940	26225990	1461	85.92	163	18	0.234	0.043	70.5	140.045	0.986	4.861
TAL3.4	8600	6850	9953623	1453	79.65	453	302	5.778	0.861	492.943	531.912	0.844	41.559
TAL3.5	16217	14040	20524637	1461	86.58	136	19	0.287	0.054	64.5	66.733	0.987	5.709
TAL3.6	9069	7800	11232088	1440	86.01	435	196	2.942	0.486	470.029	536.26	0.868	35.53
TAM1.1	10371	8489	12402136	1460	81.85	306	102	1.437	0.25	280.125	282.124	0.928	19.97
TAM1.2	8769	7556	11027753	1459	86.17	314	175	3.495	0.596	258.217	280.74	0.916	28.579
TAM1.3	9456	6729	9829151	1460	71.16	360	263	5.97	0.904	347.792	378.538	0.894	36.363
TAM1.4	11008	9495	13876260	1461	86.26	253	61	0.949	0.176	176	265.652	0.956	13.099
TAM1.5	9198	7549	11019735	1459	82.07	419	175	2.794	0.471	367.794	388.722	0.891	30.394
TAM2.1	23454	20805	30414532	1461	88.71	151	17	0.207	0.038	122	207.105	0.986	5.656
TAM2.2	15557	13687	20010069	1461	87.98	172	26	0.312	0.056	302	523.241	0.977	9.414
TAM2.3	15347	12657	18504132	1461	82.47	252	43	0.595	0.107	113.857	124.46	0.97	12.448
TAM2.4	25772	21542	31495348	1462	83.59	172	15	0.248	0.049	30	54.138	0.99	5.247
TAM2.5	20282	16837	24614306	1461	83.01	202	22	0.304	0.056	67.333	97.642	0.984	6.235
TAM2.6	7923	6444	9395252	1457	81.33	437	339	7.372	0.982	461.802	516.165	0.857	45.212
TAM3.1	6500	5383	7856790	1459	82.82	394	304	7.043	0.977	407.909	433.418	0.879	41.44
TAM3.2	7960	6456	9435426	1461	81.11	439	336	7.831	0.993	409.652	436.897	0.891	44.807

TAM3.3	6408	5049	7372945	1460	78.79	344	271	6.686	0.955	333.466	347.978	0.909	40.36
TAM3.4	11190	9774	14268207	1459	87.35	731	411	8.019	0.993	633.645	654.634	0.806	48.59
TAM3.5	28588	24340	35581042	1461	85.14	272	25	0.317	0.058	140.5	304.264	0.979	8.841
TAM3.6	7026	6043	8820295	1459	86.01	433	338	7.602	0.99	447.6	473.669	0.87	44.668
TAH1.1	11868	10575	15443905	1460	89.11	314	81	1.214	0.218	247.364	292.884	0.942	18.058
TAH1.2	8914	6886	10043213	1458	77.25	345	185	2.946	0.499	353.837	368.417	0.885	32.312
TAH1.3	6265	5481	8004507	1460	87.49	223	94	1.425	0.253	193.048	260.455	0.938	19.079
TAH1.4	7883	6899	10060749	1458	87.52	413	322	6.961	0.963	461.57	501.275	0.858	43.266
TAH1.5	11486	10320	15069407	1460	89.85	309	88	1.292	0.229	232	236.071	0.939	17.034
TAH2.1	16892	14874	21745378	1461	88.05	126	16	0.205	0.038	55	98.873	0.988	6.522
TAH2.2	23551	20927	30595212	1461	88.86	101	11	0.156	0.03	47	85.571	0.991	3.74
TAH2.3	20581	17199	25145565	1462	83.57	91	12	0.16	0.03	57	101.444	0.99	3.674
TAH2.4	21532	18061	26403031	1461	83.88	149	21	0.277	0.051	89	107.962	0.984	7.794
TAH2.5	22114	19404	28367115	1461	87.75	152	14	0.204	0.039	41.5	92.287	0.99	5.158
TAH2.6	42053	34719	50748839	1461	82.56	250	22	0.271	0.049	107.5	194.737	0.982	7.283
TAH3.1	13489	10398	15161722	1458	77.09	690	402	7.952	0.992	644.701	712.961	0.804	49.051
TAH3.3	7567	5956	8690819	1459	78.71	265	214	6.783	0.984	234.811	257.272	0.947	30.746
TAH3.4	10392	8593	12530781	1458	82.69	640	406	7.97	0.993	611.03	678.307	0.807	52.623
TAH3.5	8488	6895	10064858	1459	81.23	416	315	7.414	0.985	418.054	427.073	0.882	42.067
TAH3.6	12743	10851	15818957	1457	85.15	699	424	7.973	0.991	704	744.203	0.786	50.592
