# Supplementary material for: Tannic acid supplementation in the diet of Holstein bulls: Impacts on production performance, physiological and immunological characteristics, and ruminal microbiota
Source: Front Nutr. 2022 Nov 16;9:1066074. doi: 10.3389/fnut.2022.1066074 (PMC9709124; doi:10.3389/fnut.2022.1066074)
Supplement: Supplementary file 7 [file Table_2.PDF]

Supplementary Material

Tannic acid supplementation in the diet of Holstein bulls: Impacts on production performance, physiological and immunological characteristics, and ruminal microbiota

Zuo Wang [1], Yuan Zhao [1], Xinyi Lan [1], Jianhua He [1], Fachun Wan [1]*, Weijun Shen [1]*, Shaoxun Tang [2], Chuanshe Zhou [2], Zhiliang Tan [2], Yanming Yang [3]

[1] College of Animal Science and Technology, Hunan Agricultural University, Changsha, Hunan 410128, China

[2] CAS Key Laboratory of Agro-Ecological Processes in Subtropical Region, National Engineering Laboratory for Pollution Control and Waste Utilization in Livestock and Poultry Production, Hunan Provincial Key Laboratory of Animal Nutrition & Physiology and Metabolism, Institute of Subtropical Agriculture, Chinese Academy of Sciences, Changsha, Hunan 410125, China

[3] Jiurui Biology & Chemistry Co. Ltd., Zhangjiajie, Hunan 427000, China

*** Correspondence:**

Fachun Wan; Weijun Shen

wanfc@sina.com; shenweijun@hunau.edu.cn

Supplementary Tables

Table S2. t-test analysis for KEGG pathways

CON VS. TAL								
Taxa	avg(CON)	sd(CON)	avg(TAL)	sd(TAL)	p.value	q.values	interval lower	interval upper
Metabolism;Carbohydrate metabolism	0.106575	0.006354	0.10903	0.005088	0.209849	0.054133	-0.00636	0.001451
Environmental Information Processing;Membrane transport	0.100551	0.012099	0.096713	0.011145	0.329195	0.068158	-0.00404	0.01172
Genetic Information Processing;Replication and repair	0.093262	0.011123	0.09837	0.008323	0.12873	0.04377	-0.01178	0.001566
Genetic Information Processing;Translation	0.090686	0.007792	0.095405	0.007018	0.064792	0.04377	-0.00974	0.000306
Metabolism;Amino acid metabolism	0.089946	0.003092	0.090449	0.00294	0.620241	0.089332	-0.00255	0.001541
Metabolism;Energy metabolism	0.045583	0.000879	0.045177	0.001355	0.294852	0.06572	-0.00037	0.001185
Metabolism;Nucleotide metabolism	0.04082	0.00477	0.043087	0.003808	0.124748	0.04377	-0.0052	0.000662
Metabolism;Glycan biosynthesis and metabolism	0.034937	0.00355	0.03559	0.004088	0.612375	0.089332	-0.00325	0.001943
Metabolism;Metabolism of cofactors and vitamins	0.032773	0.000537	0.032934	0.000511	0.363944	0.071289	-0.00052	0.000194
Environmental Information Processing;Signal transduction	0.031659	0.005182	0.02882	0.005296	0.1132	0.04377	-0.00071	0.006389
Cellular Processes;Transport and catabolism	0.026372	0.002298	0.026951	0.002186	0.444256	0.077697	-0.0021	0.000941
Metabolism;Lipid metabolism	0.026577	0.003587	0.024924	0.002582	0.122726	0.04377	-0.00047	0.003778
Metabolism;Enzyme families	0.025403	0.001184	0.025248	0.001307	0.711638	0.091069	-0.00069	0.001
Genetic Information Processing;Folding, sorting and degradation	0.024766	0.001364	0.024871	0.001313	0.815046	0.095962	-0.00101	0.000802
Cellular Processes;Cell motility	0.022571	0.004318	0.019895	0.005735	0.123661	0.04377	-0.00077	0.006125
Cellular Processes;Cellular community - prokaryotes	0.021276	0.00392	0.019175	0.002901	0.077091	0.04377	-0.00024	0.004444
Genetic Information Processing;Transcription	0.018513	0.00046	0.018478	0.000469	0.823041	0.095962	-0.00028	0.000349
Unclassified;Metabolism	0.016072	0.001039	0.01595	0.00086	0.70393	0.091069	-0.00052	0.000769

Unclassified;Cellular processes and signaling	0.015858	0.001002	0.016026	0.000847	0.589847	0.089332	-0.0008	0.000461
Metabolism;Metabolism of other amino acids	0.015639	0.001468	0.015494	0.000906	0.725283	0.091069	-0.00069	0.000977
Metabolism;Biosynthesis of other secondary metabolites	0.013805	0.00092	0.014083	0.001077	0.409905	0.074344	-0.00096	0.000401
Unclassified;Genetic information processing	0.012553	0.000948	0.012661	0.001101	0.75428	0.092342	-0.0008	0.000588
Metabolism;Xenobiotics biodegradation and metabolism	0.012623	0.004741	0.010782	0.00385	0.210033	0.054133	-0.00109	0.004771
Metabolism;Metabolism of terpenoids and polyketides	0.011426	0.001474	0.01098	0.000945	0.288317	0.06572	-0.0004	0.00129
Human Diseases;Drug resistance	0.011265	0.000684	0.010964	0.00066	0.188532	0.054133	-0.00015	0.000756
Unclassified;Poorly characterized	0.009923	0.000285	0.010104	0.000385	0.11791	0.04377	-0.00041	4.87E-05
Cellular Processes;Cell growth and death	0.009183	0.000989	0.009234	0.000977	0.876103	0.097505	-0.00072	0.000614
Organismal Systems;Endocrine system	0.008027	0.000497	0.007666	0.000562	0.048865	0.04377	1.91E-06	0.000721
Human Diseases;Infectious diseases	0.006803	0.000376	0.006702	0.000224	0.334042	0.068158	-0.00011	0.000313
Human Diseases;Cancers	0.005042	0.000311	0.005224	0.000258	0.065402	0.04377	-0.00037	1.22E-05
Organismal Systems;Aging	0.003371	0.000233	0.003342	0.000136	0.652002	0.089434	-0.0001	0.000159
Human Diseases;Endocrine and metabolic diseases	0.002839	0.000214	0.002908	0.000176	0.295253	0.06572	-0.0002	6.34E-05
Organismal Systems;Nervous system	0.002678	0.000508	0.00282	0.000444	0.379112	0.071404	-0.00047	0.000182
Organismal Systems;Immune system	0.002773	0.000469	0.002679	0.000424	0.531975	0.086835	-0.00021	0.000397
Organismal Systems;Environmental adaptation	0.001467	0.000167	0.001376	0.000203	0.152581	0.046699	-3.5E-05	0.000217
Environmental Information Processing;Signaling molecules and interaction	0.001428	0.000314	0.00136	0.000327	0.528301	0.086835	-0.00015	0.000285
Human Diseases;Cardiovascular diseases	0.001393	0.000149	0.001302	0.000115	0.049266	0.04377	3.1E-07	0.000181
Human Diseases;Neurodegenerative diseases	0.001302	0.000589	0.001032	0.000415	0.121835	0.04377	-7.6E-05	0.000617

Organismal Systems;Digestive system	0.000774	0.000333	0.000755	0.000372	0.868618	0.097505	-0.00022	0.000259
Unclassified;Viral protein family	0.000644	7.95E-05	0.000706	6.94E-05	0.017808	0.04377	-0.00011	-1.1E-05
Human Diseases;Immune diseases	0.000318	3.69E-05	0.000309	4.73E-05	0.555962	0.087823	-2E-05	3.72E-05
Organismal Systems;Excretory system	0.00026	9.84E-05	0.000247	7.96E-05	0.657471	0.089434	-4.7E-05	7.41E-05
Organismal Systems;Circulatory system	0.000165	0.000156	9.59E-05	0.000109	0.134073	0.04377	-2.2E-05	0.00016
Human Diseases;Substance dependence	9.75E-05	2.93E-05	8.03E-05	3.08E-05	0.096465	0.04377	-3.2E-06	3.75E-05
CON VS. TAM								
Taxa	avg(CON)	sd(CON)	avg(TAM)	sd(TAM)	p.value	q.values	interval lower	interval upper
Metabolism;Carbohydrate metabolism	0.106575	0.006354	0.107516	0.005494	0.641974	0.619522	-0.00502	0.003139
Environmental Information Processing;Membrane transport	0.100551	0.012099	0.099418	0.009871	0.762685	0.662235	-0.00645	0.008715
Genetic Information Processing;Replication and repair	0.093262	0.011123	0.095317	0.010065	0.570139	0.619522	-0.00934	0.005234
Genetic Information Processing;Translation	0.090686	0.007792	0.091911	0.007699	0.642856	0.619522	-0.00655	0.004103
Metabolism;Amino acid metabolism	0.089946	0.003092	0.089987	0.002879	0.967471	0.706405	-0.0021	0.002012
Metabolism;Energy metabolism	0.045583	0.000879	0.045094	0.000987	0.13233	0.604558	-0.00016	0.001134
Metabolism;Nucleotide metabolism	0.04082	0.00477	0.041541	0.004539	0.649805	0.619522	-0.00392	0.00248
Metabolism;Glycan biosynthesis and metabolism	0.034937	0.00355	0.033904	0.004071	0.430764	0.619522	-0.0016	0.00367
Metabolism;Metabolism of cofactors and vitamins	0.032773	0.000537	0.03243	0.000511	0.061482	0.604558	-1.7E-05	0.000703
Environmental Information Processing;Signal transduction	0.031659	0.005182	0.031988	0.00571	0.859675	0.690467	-0.00409	0.003431
Cellular Processes;Transport and catabolism	0.026372	0.002298	0.026219	0.00207	0.837385	0.689809	-0.00135	0.001655
Metabolism;Lipid metabolism	0.026577	0.003587	0.025339	0.003194	0.288135	0.604558	-0.0011	0.003571
Metabolism;Enzyme families	0.025403	0.001184	0.025246	0.001158	0.693988	0.637019	-0.00065	0.000963

Genetic Information Processing;Folding, sorting and degradation	0.024766	0.001364	0.024415	0.00164	0.498531	0.619522	-0.00069	0.001393
Cellular Processes;Cell motility	0.022571	0.004318	0.024636	0.006441	0.277695	0.604558	-0.00589	0.001756
Cellular Processes;Cellular community - prokaryotes	0.021276	0.00392	0.020356	0.003619	0.475247	0.619522	-0.00167	0.003513
Genetic Information Processing;Transcription	0.018513	0.00046	0.018734	0.00047	0.170542	0.604558	-0.00054	9.97E-05
Unclassified;Metabolism	0.016072	0.001039	0.015975	0.00078	0.756277	0.662235	-0.00053	0.000727
Unclassified;Cellular processes and signaling	0.015858	0.001002	0.015973	0.000388	0.655642	0.619522	-0.00064	0.000412
Metabolism;Metabolism of other amino acids	0.015639	0.001468	0.015164	0.00107	0.280903	0.604558	-0.00041	0.001357
Metabolism;Biosynthesis of other secondary metabolites	0.013805	0.00092	0.013834	0.000727	0.9155	0.701126	-0.0006	0.000539
Unclassified;Genetic information processing	0.012553	0.000948	0.013372	0.001966	0.133943	0.604558	-0.00191	0.000272
Metabolism;Xenobiotics biodegradation and metabolism	0.012623	0.004741	0.011758	0.004372	0.578081	0.619522	-0.00227	0.004
Metabolism;Metabolism of terpenoids and polyketides	0.011426	0.001474	0.010945	0.001235	0.301573	0.604558	-0.00045	0.001415
Human Diseases;Drug resistance	0.011265	0.000684	0.01092	0.000778	0.174582	0.604558	-0.00016	0.00085
Unclassified;Poorly characterized	0.009923	0.000285	0.009834	0.000276	0.357538	0.604558	-0.0001	0.000281
Cellular Processes;Cell growth and death	0.009183	0.000989	0.009168	0.000852	0.962317	0.706405	-0.00062	0.000649
Organismal Systems;Endocrine system	0.008027	0.000497	0.007861	0.000516	0.339569	0.604558	-0.00018	0.000515
Human Diseases;Infectious diseases	0.006803	0.000376	0.006674	0.000299	0.269018	0.604558	-0.0001	0.000362
Human Diseases;Cancers	0.005042	0.000311	0.005156	0.000246	0.239256	0.604558	-0.00031	7.91E-05
Organismal Systems;Aging	0.003371	0.000233	0.003278	0.000179	0.193988	0.604558	-5E-05	0.000236
Human Diseases;Endocrine and metabolic diseases	0.002839	0.000214	0.002855	0.000188	0.811212	0.685835	-0.00015	0.000122
Organismal Systems;Nervous system	0.002678	0.000508	0.002822	0.00036	0.341347	0.604558	-0.00045	0.000159

Organismal Systems;Immune system	0.002773	0.000469	0.002833	0.000272	0.646836	0.619522	-0.00032	0.000204
Organismal Systems;Environmental adaptation	0.001467	0.000167	0.001521	9.39E-05	0.247298	0.604558	-0.00015	3.96E-05
Environmental Information Processing;Signaling molecules and interaction	0.001428	0.000314	0.001365	0.000367	0.589379	0.619522	-0.00017	0.000299
Human Diseases;Cardiovascular diseases	0.001393	0.000149	0.001344	0.000154	0.341152	0.604558	-5.5E-05	0.000154
Human Diseases;Neurodegenerative diseases	0.001302	0.000589	0.001146	0.00054	0.417022	0.619522	-0.00023	0.000545
Organismal Systems;Digestive system	0.000774	0.000333	0.000688	0.000349	0.458889	0.619522	-0.00015	0.000322
Unclassified;Viral protein family	0.000644	7.95E-05	0.00068	7.65E-05	0.187056	0.604558	-8.9E-05	1.81E-05
Human Diseases;Immune diseases	0.000318	3.69E-05	0.000297	6.21E-05	0.251823	0.604558	-1.5E-05	5.61E-05
Organismal Systems;Excretory system	0.00026	9.84E-05	0.000264	9.43E-05	0.916594	0.701126	-7E-05	6.28E-05
Organismal Systems;Circulatory system	0.000165	0.000156	0.000122	0.00014	0.40052	0.619522	-5.9E-05	0.000144
Human Diseases;Substance dependence	9.75E-05	2.93E-05	0.000102	2.35E-05	0.642178	0.619522	-2.2E-05	1.4E-05
CON VS. TAH								
Taxa	avg(CON)	sd(CON)	avg(TAH)	sd(TAH)	p.value	q.values	interval lower	interval upper
Metabolism;Carbohydrate metabolism	0.106575	0.006354	0.107426	0.006171	0.695042	1	-0.00523	0.00353
Environmental Information Processing;Membrane transport	0.100551	0.012099	0.09835	0.011443	0.589595	1	-0.00603	0.010431
Genetic Information Processing;Replication and repair	0.093262	0.011123	0.094718	0.010178	0.692826	1	-0.0089	0.005986
Genetic Information Processing;Translation	0.090686	0.007792	0.09224	0.007491	0.557684	1	-0.0069	0.00379
Metabolism;Amino acid metabolism	0.089946	0.003092	0.090428	0.002376	0.611746	1	-0.0024	0.001435
Metabolism;Energy metabolism	0.045583	0.000879	0.045723	0.000982	0.665443	1	-0.0008	0.000516
Metabolism;Nucleotide metabolism	0.04082	0.00477	0.04148	0.004447	0.67912	1	-0.00388	0.002561
Metabolism;Glycan biosynthesis and metabolism	0.034937	0.00355	0.035522	0.003818	0.64822	1	-0.00317	0.002005

Metabolism;Metabolism of cofactors and vitamins	0.032773	0.000537	0.032847	0.000504	0.679783	1	-0.00044	0.000289
Environmental Information Processing;Signal transduction	0.031659	0.005182	0.030427	0.005233	0.496004	1	-0.00242	0.004881
Cellular Processes;Transport and catabolism	0.026372	0.002298	0.026658	0.002138	0.709742	1	-0.00184	0.001264
Metabolism;Lipid metabolism	0.026577	0.003587	0.026	0.003252	0.625923	1	-0.00181	0.002967
Metabolism;Enzyme families	0.025403	0.001184	0.025513	0.001244	0.794113	1	-0.00096	0.000742
Genetic Information Processing;Folding, sorting and degradation	0.024766	0.001364	0.024817	0.001285	0.910591	1	-0.00098	0.000875
Cellular Processes;Cell motility	0.022571	0.004318	0.021416	0.00492	0.474826	1	-0.0021	0.004415
Cellular Processes;Cellular community - prokaryotes	0.021276	0.00392	0.020672	0.003742	0.648927	1	-0.00207	0.003283
Genetic Information Processing;Transcription	0.018513	0.00046	0.018434	0.000448	0.612746	1	-0.00024	0.000397
Unclassified;Metabolism	0.016072	0.001039	0.016097	0.000899	0.942179	1	-0.0007	0.000653
Unclassified;Cellular processes and signaling	0.015858	0.001002	0.015949	0.000798	0.770445	1	-0.00072	0.000539
Metabolism;Metabolism of other amino acids	0.015639	0.001468	0.015596	0.001174	0.926126	1	-0.00088	0.000967
Metabolism;Biosynthesis of other secondary metabolites	0.013805	0.00092	0.01402	0.000905	0.497031	1	-0.00085	0.000423
Unclassified;Genetic information processing	0.012553	0.000948	0.012451	0.001071	0.771774	1	-0.00061	0.000814
Metabolism;Xenobiotics biodegradation and metabolism	0.012623	0.004741	0.011996	0.004621	0.698934	1	-0.00265	0.003902
Metabolism;Metabolism of terpenoids and polyketides	0.011426	0.001474	0.011408	0.001357	0.969591	1	-0.00097	0.001008
Human Diseases;Drug resistance	0.011265	0.000684	0.011175	0.000725	0.714375	1	-0.00041	0.000584
Unclassified;Poorly characterized	0.009923	0.000285	0.009929	0.000175	0.938467	1	-0.00017	0.000158
Cellular Processes;Cell growth and death	0.009183	0.000989	0.009301	0.000911	0.71954	1	-0.00078	0.000546

Organismal Systems;Endocrine system	0.008027	0.000497	0.007963	0.00046	0.695772	1	-0.00027	0.000399
Human Diseases;Infectious diseases	0.006803	0.000376	0.006798	0.000348	0.967055	1	-0.00025	0.000258
Human Diseases;Cancers	0.005042	0.000311	0.005077	0.000297	0.741026	1	-0.00025	0.000178
Organismal Systems;Aging	0.003371	0.000233	0.003384	0.000169	0.854728	1	-0.00015	0.000129
Human Diseases;Endocrine and metabolic diseases	0.002839	0.000214	0.00289	0.000162	0.432276	1	-0.00018	8.04E-05
Organismal Systems;Nervous system	0.002678	0.000508	0.002758	0.000506	0.649493	1	-0.00044	0.000275
Organismal Systems;Immune system	0.002773	0.000469	0.002775	0.000358	0.988951	1	-0.00029	0.000288
Organismal Systems;Environmental adaptation	0.001467	0.000167	0.00142	0.000155	0.400886	1	-6.6E-05	0.00016
Environmental Information Processing;Signaling molecules and interaction	0.001428	0.000314	0.001486	0.000305	0.586541	1	-0.00027	0.000158
Human Diseases;Cardiovascular diseases	0.001393	0.000149	0.001368	0.000153	0.627386	1	-8E-05	0.000131
Human Diseases;Neurodegenerative diseases	0.001302	0.000589	0.001222	0.000556	0.684642	1	-0.00032	0.000481
Organismal Systems;Digestive system	0.000774	0.000333	0.000806	0.000366	0.794467	1	-0.00028	0.000215
Unclassified;Viral protein family	0.000644	7.95E-05	0.000653	8.28E-05	0.744987	1	-6.6E-05	4.78E-05
Human Diseases;Immune diseases	0.000318	3.69E-05	0.000313	3.44E-05	0.737305	1	-2.1E-05	2.91E-05
Organismal Systems;Excretory system	0.00026	9.84E-05	0.000255	7.7E-05	0.86883	1	-5.6E-05	6.65E-05
Organismal Systems;Circulatory system	0.000165	0.000156	0.000143	0.000147	0.675482	1	-8.4E-05	0.000128
Human Diseases;Substance dependence	9.75E-05	2.93E-05	9.69E-05	3.56E-05	0.956975	1	-2.2E-05	2.36E-05
TAL VS. TAM								
Taxa	avg(TAL)	sd(TAL)	avg(TAM)	sd(TAM)	p.value	q.values	interval lower	interval upper
Metabolism;Carbohydrate metabolism	0.10903	0.005088	0.107516	0.005494	0.40464	0.794338	-0.00214	0.005164
Environmental Information Processing;Membrane transport	0.096713	0.011145	0.099418	0.009871	0.452076	0.803234	-0.00994	0.004527

Genetic Information Processing;Replication and repair	0.09837	0.008323	0.095317	0.010065	0.337069	0.794338	-0.00333	0.00944
Genetic Information Processing;Translation	0.095405	0.007018	0.091911	0.007699	0.170949	0.794338	-0.00159	0.008573
Metabolism;Amino acid metabolism	0.090449	0.00294	0.089987	0.002879	0.642102	0.898865	-0.00154	0.002463
Metabolism;Energy metabolism	0.045177	0.001355	0.045094	0.000987	0.837238	1	-0.00073	0.000897
Metabolism;Nucleotide metabolism	0.043087	0.003808	0.041541	0.004539	0.284629	0.794338	-0.00135	0.004442
Metabolism;Glycan biosynthesis and metabolism	0.03559	0.004088	0.033904	0.004071	0.230443	0.794338	-0.00112	0.004493
Metabolism;Metabolism of cofactors and vitamins	0.032934	0.000511	0.03243	0.000511	0.006377	0.262748	0.000152	0.000856
Environmental Information Processing;Signal transduction	0.02882	0.005296	0.031988	0.00571	0.098786	0.760587	-0.00696	0.000627
Cellular Processes;Transport and catabolism	0.026951	0.002186	0.026219	0.00207	0.316671	0.794338	-0.00073	0.002195
Metabolism;Lipid metabolism	0.024924	0.002582	0.025339	0.003194	0.676489	0.91915	-0.00242	0.001595
Metabolism;Enzyme families	0.025248	0.001307	0.025246	0.001158	0.995813	1	-0.00085	0.00085
Genetic Information Processing;Folding, sorting and degradation	0.024871	0.001313	0.024415	0.00164	0.373225	0.794338	-0.00057	0.001484
Cellular Processes;Cell motility	0.019895	0.005735	0.024636	0.006441	0.028438	0.262748	-0.00895	-0.00053
Cellular Processes;Cellular community - prokaryotes	0.019175	0.002901	0.020356	0.003619	0.296829	0.794338	-0.00345	0.001089
Genetic Information Processing;Transcription	0.018478	0.000469	0.018734	0.00047	0.117526	0.775608	-0.00058	6.79E-05
Unclassified;Metabolism	0.01595	0.00086	0.015975	0.00078	0.928705	1	-0.00059	0.000539
Unclassified;Cellular processes and signaling	0.016026	0.000847	0.015973	0.000388	0.810835	1	-0.0004	0.000509
Metabolism;Metabolism of other amino acids	0.015494	0.000906	0.015164	0.00107	0.333149	0.794338	-0.00035	0.001015
Metabolism;Biosynthesis of other secondary metabolites	0.014083	0.001077	0.013834	0.000727	0.427161	0.794338	-0.00038	0.00088
Unclassified;Genetic information processing	0.012661	0.001101	0.013372	0.001966	0.20268	0.794338	-0.00183	0.000408

Metabolism;Xenobiotics biodegradation and metabolism	0.010782	0.00385	0.011758	0.004372	0.489776	0.812883	-0.00382	0.001868
Metabolism;Metabolism of terpenoids and polyketides	0.01098	0.000945	0.010945	0.001235	0.925645	1	-0.00073	0.000798
Human Diseases;Drug resistance	0.010964	0.00066	0.01092	0.000778	0.858109	1	-0.00045	0.000543
Unclassified;Poorly characterized	0.010104	0.000385	0.009834	0.000276	0.022935	0.262748	3.99E-05	0.0005
Cellular Processes;Cell growth and death	0.009234	0.000977	0.009168	0.000852	0.831697	1	-0.00056	0.000696
Organismal Systems;Endocrine system	0.007666	0.000562	0.007861	0.000516	0.291351	0.794338	-0.00057	0.000175
Human Diseases;Infectious diseases	0.006702	0.000224	0.006674	0.000299	0.762218	1	-0.00016	0.000211
Human Diseases;Cancers	0.005224	0.000258	0.005156	0.000246	0.429874	0.794338	-0.00011	0.000241
Organismal Systems;Aging	0.003342	0.000136	0.003278	0.000179	0.245771	0.794338	-4.6E-05	0.000174
Human Diseases;Endocrine and metabolic diseases	0.002908	0.000176	0.002855	0.000188	0.396278	0.794338	-7.3E-05	0.000179
Organismal Systems;Nervous system	0.00282	0.000444	0.002822	0.00036	0.990981	1	-0.00028	0.000276
Organismal Systems;Immune system	0.002679	0.000424	0.002833	0.000272	0.209402	0.794338	-0.0004	9.12E-05
Organismal Systems;Environmental adaptation	0.001376	0.000203	0.001521	9.39E-05	0.011562	0.262748	-0.00025	-3.5E-05
Environmental Information Processing;Signaling molecules and interaction	0.00136	0.000327	0.001365	0.000367	0.966445	1	-0.00024	0.000235
Human Diseases;Cardiovascular diseases	0.001302	0.000115	0.001344	0.000154	0.379512	0.794338	-0.00014	5.31E-05
Human Diseases;Neurodegenerative diseases	0.001032	0.000415	0.001146	0.00054	0.492699	0.812883	-0.00045	0.00022
Organismal Systems;Digestive system	0.000755	0.000372	0.000688	0.000349	0.586753	0.847052	-0.00018	0.000315
Unclassified;Viral protein family	0.000706	6.94E-05	0.00068	7.65E-05	0.292248	0.794338	-2.4E-05	7.68E-05
Human Diseases;Immune diseases	0.000309	4.73E-05	0.000297	6.21E-05	0.527965	0.834743	-2.6E-05	5.02E-05
Organismal Systems;Excretory system	0.000247	7.96E-05	0.000264	9.43E-05	0.574261	0.847052	-7.7E-05	4.35E-05
Organismal Systems;Circulatory system	9.59E-05	0.000109	0.000122	0.00014	0.542088	0.834743	-0.00011	6.07E-05

Human Diseases;Substance dependence	8.03E-05	3.08E-05	0.000102	2.35E-05	0.027577	0.262748	-4E-05	-2.5E-06
TAL VS. TAH								
Taxa	avg(TAL)	sd(TAL)	avg(TAH)	sd(TAH)	p.value	q.values	interval lower	interval upper
Metabolism;Carbohydrate metabolism	0.10903	0.005088	0.107426	0.006171	0.418387	0.529381	-0.00239	0.005599
Environmental Information Processing;Membrane transport	0.096713	0.011145	0.09835	0.011443	0.676188	0.561591	-0.00956	0.00628
Genetic Information Processing;Replication and repair	0.09837	0.008323	0.094718	0.010178	0.264978	0.529095	-0.00292	0.010223
Genetic Information Processing;Translation	0.095405	0.007018	0.09224	0.007491	0.214753	0.529095	-0.00193	0.008261
Metabolism;Amino acid metabolism	0.090449	0.00294	0.090428	0.002376	0.981938	0.622053	-0.00184	0.001881
Metabolism;Energy metabolism	0.045177	0.001355	0.045723	0.000982	0.184853	0.529095	-0.00137	0.000275
Metabolism;Nucleotide metabolism	0.043087	0.003808	0.04148	0.004447	0.269695	0.529095	-0.00131	0.004526
Metabolism;Glycan biosynthesis and metabolism	0.03559	0.004088	0.035522	0.003818	0.960399	0.622053	-0.0027	0.002831
Metabolism;Metabolism of cofactors and vitamins	0.032934	0.000511	0.032847	0.000504	0.623614	0.561591	-0.00027	0.000442
Environmental Information Processing;Signal transduction	0.02882	0.005296	0.030427	0.005233	0.380928	0.529381	-0.00529	0.002078
Cellular Processes;Transport and catabolism	0.026951	0.002186	0.026658	0.002138	0.695915	0.561591	-0.00122	0.001806
Metabolism;Lipid metabolism	0.024924	0.002582	0.026	0.003252	0.298393	0.529095	-0.00315	0.001003
Metabolism;Enzyme families	0.025248	0.001307	0.025513	0.001244	0.549263	0.561591	-0.00116	0.000627
Genetic Information Processing;Folding, sorting and degradation	0.024871	0.001313	0.024817	0.001285	0.904842	0.600509	-0.00086	0.000963
Cellular Processes;Cell motility	0.019895	0.005735	0.021416	0.00492	0.411358	0.529381	-0.00524	0.002202
Cellular Processes;Cellular community - prokaryotes	0.019175	0.002901	0.020672	0.003742	0.206879	0.529095	-0.00387	0.000876
Genetic Information Processing;Transcription	0.018478	0.000469	0.018434	0.000448	0.777912	0.561591	-0.00028	0.000365

Unclassified;Metabolism	0.01595	0.00086	0.016097	0.000899	0.632484	0.561591	-0.00076	0.000471
Unclassified;Cellular processes and signaling	0.016026	0.000847	0.015949	0.000798	0.785758	0.561591	-0.0005	0.000652
Metabolism;Metabolism of other amino acids	0.015494	0.000906	0.015596	0.001174	0.78098	0.561591	-0.00085	0.000641
Metabolism;Biosynthesis of other secondary metabolites	0.014083	0.001077	0.01402	0.000905	0.853494	0.580247	-0.00063	0.000756
Unclassified;Genetic information processing	0.012661	0.001101	0.012451	0.001071	0.576996	0.561591	-0.00055	0.00097
Metabolism;Xenobiotics biodegradation and metabolism	0.010782	0.00385	0.011996	0.004621	0.415492	0.529381	-0.00422	0.00179
Metabolism;Metabolism of terpenoids and polyketides	0.01098	0.000945	0.011408	0.001357	0.301626	0.529095	-0.00126	0.000406
Human Diseases;Drug resistance	0.010964	0.00066	0.011175	0.000725	0.383935	0.529381	-0.0007	0.000276
Unclassified;Poorly characterized	0.010104	0.000385	0.009929	0.000175	0.094716	0.529095	-3.3E-05	0.000383
Cellular Processes;Cell growth and death	0.009234	0.000977	0.009301	0.000911	0.838405	0.580247	-0.00073	0.000593
Organismal Systems;Endocrine system	0.007666	0.000562	0.007963	0.00046	0.100568	0.529095	-0.00065	6.08E-05
Human Diseases;Infectious diseases	0.006702	0.000224	0.006798	0.000348	0.353027	0.529381	-0.00031	0.000113
Human Diseases;Cancers	0.005224	0.000258	0.005077	0.000297	0.137507	0.529095	-5E-05	0.000343
Organismal Systems;Aging	0.003342	0.000136	0.003384	0.000169	0.436818	0.529381	-0.00015	6.67E-05
Human Diseases;Endocrine and metabolic diseases	0.002908	0.000176	0.00289	0.000162	0.758584	0.561591	-0.0001	0.000136
Organismal Systems;Nervous system	0.00282	0.000444	0.002758	0.000506	0.70944	0.561591	-0.00027	0.000397
Organismal Systems;Immune system	0.002679	0.000424	0.002775	0.000358	0.479245	0.538368	-0.00037	0.000177
Organismal Systems;Environmental adaptation	0.001376	0.000203	0.00142	0.000155	0.482862	0.538368	-0.00017	8.18E-05
Environmental Information Processing;Signaling molecules and interaction	0.00136	0.000327	0.001486	0.000305	0.251786	0.529095	-0.00035	9.43E-05
Human Diseases;Cardiovascular diseases	0.001302	0.000115	0.001368	0.000153	0.174557	0.529095	-0.00016	3.07E-05

Human Diseases;Neurodegenerative diseases	0.001032	0.000415	0.001222	0.000556	0.274133	0.529095	-0.00054	0.000159
Organismal Systems;Digestive system	0.000755	0.000372	0.000806	0.000366	0.688412	0.561591	-0.00031	0.000207
Unclassified;Viral protein family	0.000706	6.94E-05	0.000653	8.28E-05	0.054499	0.529095	-1.1E-06	0.000107
Human Diseases;Immune diseases	0.000309	4.73E-05	0.000313	3.44E-05	0.763422	0.561591	-3.3E-05	2.45E-05
Organismal Systems;Excretory system	0.000247	7.96E-05	0.000255	7.7E-05	0.758663	0.561591	-6.3E-05	4.65E-05
Organismal Systems;Circulatory system	9.59E-05	0.000109	0.000143	0.000147	0.303709	0.529095	-0.00014	4.49E-05
Human Diseases;Substance dependence	8.03E-05	3.08E-05	9.69E-05	3.56E-05	0.160555	0.529095	-4E-05	6.93E-06
TAM VS. TAH								
Taxa	avg(TAM)	sd(TAM)	avg(TAH)	sd(TAH)	p.value	q.values	interval lower	interval upper
Metabolism;Carbohydrate metabolism	0.107516	0.005494	0.107426	0.006171	0.965017	0.742057	-0.00407	0.004253
Environmental Information Processing;Membrane transport	0.099418	0.009871	0.09835	0.011443	0.776779	0.709386	-0.00655	0.008689
Genetic Information Processing;Replication and repair	0.095317	0.010065	0.094718	0.010178	0.866351	0.733586	-0.0066	0.007792
Genetic Information Processing;Translation	0.091911	0.007699	0.09224	0.007491	0.901961	0.733586	-0.00572	0.005065
Metabolism;Amino acid metabolism	0.089987	0.002879	0.090428	0.002376	0.634226	0.66641	-0.00231	0.001431
Metabolism;Energy metabolism	0.045094	0.000987	0.045723	0.000982	0.07615	0.557789	-0.00133	7.02E-05
Metabolism;Nucleotide metabolism	0.041541	0.004539	0.04148	0.004447	0.969217	0.742057	-0.00313	0.003252
Metabolism;Glycan biosynthesis and metabolism	0.033904	0.004071	0.035522	0.003818	0.247773	0.66641	-0.00442	0.001183
Metabolism;Metabolism of cofactors and vitamins	0.03243	0.000511	0.032847	0.000504	0.024717	0.557789	-0.00078	-5.7E-05
Environmental Information Processing;Signal transduction	0.031988	0.00571	0.030427	0.005233	0.418566	0.66641	-0.00232	0.005447
Cellular Processes;Transport and catabolism	0.026219	0.00207	0.026658	0.002138	0.554211	0.66641	-0.00193	0.001058
Metabolism;Lipid metabolism	0.025339	0.003194	0.026	0.003252	0.560612	0.66641	-0.00295	0.00163

Metabolism;Enzyme families	0.025246	0.001158	0.025513	0.001244	0.528536	0.66641	-0.00112	0.000588
Genetic Information Processing;Folding, sorting and degradation	0.024415	0.00164	0.024817	0.001285	0.438265	0.66641	-0.00145	0.000642
Cellular Processes;Cell motility	0.024636	0.006441	0.021416	0.00492	0.115904	0.557789	-0.00084	0.007282
Cellular Processes;Cellular community - prokaryotes	0.020356	0.003619	0.020672	0.003742	0.80699	0.715407	-0.00293	0.002301
Genetic Information Processing;Transcription	0.018734	0.00047	0.018434	0.000448	0.06984	0.557789	-2.6E-05	0.000626
Unclassified;Metabolism	0.015975	0.00078	0.016097	0.000899	0.68287	0.66641	-0.00072	0.000479
Unclassified;Cellular processes and signaling	0.015973	0.000388	0.015949	0.000798	0.914601	0.733586	-0.00043	0.000482
Metabolism;Metabolism of other amino acids	0.015164	0.00107	0.015596	0.001174	0.278652	0.66641	-0.00123	0.000368
Metabolism;Biosynthesis of other secondary metabolites	0.013834	0.000727	0.01402	0.000905	0.522985	0.66641	-0.00077	0.000401
Unclassified;Genetic information processing	0.013372	0.001966	0.012451	0.001071	0.104776	0.557789	-0.00021	0.002046
Metabolism;Xenobiotics biodegradation and metabolism	0.011758	0.004372	0.011996	0.004621	0.880311	0.733586	-0.00344	0.002962
Metabolism;Metabolism of terpenoids and polyketides	0.010945	0.001235	0.011408	0.001357	0.314664	0.66641	-0.00139	0.000461
Human Diseases;Drug resistance	0.01092	0.000778	0.011175	0.000725	0.337254	0.66641	-0.00079	0.000279
Unclassified;Poorly characterized	0.009834	0.000276	0.009929	0.000175	0.246609	0.66641	-0.00026	6.93E-05
Cellular Processes;Cell growth and death	0.009168	0.000852	0.009301	0.000911	0.668817	0.66641	-0.00076	0.000495
Organismal Systems;Endocrine system	0.007861	0.000516	0.007963	0.00046	0.55535	0.66641	-0.00045	0.000245
Human Diseases;Infectious diseases	0.006674	0.000299	0.006798	0.000348	0.2836	0.66641	-0.00036	0.000108
Human Diseases;Cancers	0.005156	0.000246	0.005077	0.000297	0.417222	0.66641	-0.00012	0.000273
Organismal Systems;Aging	0.003278	0.000179	0.003384	0.000169	0.090749	0.557789	-0.00023	1.78E-05
Human Diseases;Endocrine and metabolic diseases	0.002855	0.000188	0.00289	0.000162	0.569964	0.66641	-0.00016	8.96E-05

Organismal Systems;Nervous system	0.002822	0.00036	0.002758	0.000506	0.683846	0.66641	-0.00025	0.000379
Organismal Systems;Immune system	0.002833	0.000272	0.002775	0.000358	0.60836	0.66641	-0.00017	0.000285
Organismal Systems;Environmental adaptation	0.001521	9.39E-05	0.00142	0.000155	0.03397	0.557789	8.3E-06	0.000194
Environmental Information Processing;Signaling molecules and interaction	0.001365	0.000367	0.001486	0.000305	0.307665	0.66641	-0.00036	0.000117
Human Diseases;Cardiovascular diseases	0.001344	0.000154	0.001368	0.000153	0.655164	0.66641	-0.00013	8.49E-05
Human Diseases;Neurodegenerative diseases	0.001146	0.00054	0.001222	0.000556	0.692374	0.66641	-0.00047	0.000313
Organismal Systems;Digestive system	0.000688	0.000349	0.000806	0.000366	0.350485	0.66641	-0.00037	0.000136
Unclassified;Viral protein family	0.00068	7.65E-05	0.000653	8.28E-05	0.350433	0.66641	-3E-05	8.31E-05
Human Diseases;Immune diseases	0.000297	6.21E-05	0.000313	3.44E-05	0.357778	0.66641	-5.2E-05	1.95E-05
Organismal Systems;Excretory system	0.000264	9.43E-05	0.000255	7.7E-05	0.77914	0.709386	-5.3E-05	6.95E-05
Organismal Systems;Circulatory system	0.000122	0.00014	0.000143	0.000147	0.682469	0.66641	-0.00012	8.14E-05
Human Diseases;Substance dependence	0.000102	2.35E-05	9.69E-05	3.56E-05	0.652568	0.66641	-1.7E-05	2.65E-05
